# Effect of alirocumab and evolocumab on all-cause mortality and major cardiovascular events: A meta-analysis focusing on the number needed to treat

**DOI:** 10.3389/fcvm.2022.1016802

**Published:** 2022-12-02

**Authors:** Hong-Fei Wang, Yu-Cheng Mao, Xin-Yi Xu, Si-Yu Zhao, Dan-Dan Han, Shi-Yao Ge, Kai Song, Chang Geng, Qing-Bao Tian

**Affiliations:** ^1^Department of Epidemiology and Statistics, School of Public Health, Hebei Medical University, Shijiazhuang, China; ^2^Hebei Province Key Laboratory of Environment and Human Health, Shijiazhuang, China; ^3^Postdoctoral Research Station in Basic Medicine, Hebei Medical University, Shijiazhuang, China; ^4^School of Nursing, Hebei Medical University, Shijiazhuang, China

**Keywords:** PCSK 9 inhibitors, alirocumab, evolocumab, efficacy, number needed to treat (NNT)

## Abstract

**Aims:**

The efficacy of anti-proprotein convertase subtilisin/Kexin type 9 (PCSK9) monoclonal antibodies in patients with atherosclerotic cardiovascular disease (ASCVD) remains unclear. Therefore, this study aims to assess the effect of PCSK9 inhibitors (alirocumab and evolocumab) on ASCVD patients considering the number needed to treat (NNT).

**Methods:**

We reviewed randomized controlled trials (RCTs) which compared the effects of alirocumab or evolocumab and placebo or standards of care. All articles were published in English up to May 2022. Using random effect models, we estimated risk ratios (RRs), NNT, and 95% confidence intervals (CI).

**Results:**

We incorporated 12 RCTs with 53 486 patients total, of which 27 674 received PCSK9 inhibitors and 25 812 received placebos. The mean follow-up duration was 1.56 years. The effect of PCSK9 inhibitors on major adverse cardiovascular events (MACE) was statistically significant, and the corresponding mean NNT was 36. Alirocumab reduced the risk of MACE, stroke, and coronary revascularization; the corresponding mean NNT were 37, 319, and 107, respectively. Evolocumab positively affected MACE, myocardial infarction, stroke, and coronary revascularization; the corresponding mean NNT were 32, 78, 267, and 65, respectively. The effects of alirocumab or evolocumab on all-cause mortality and cardiovascular mortality were not statistically significant.

**Conclusion:**

This study suggests that preventing one patient from MACE needed to treat 36 patients with ASCVD with PCSK9 inhibitors for 1.56 years. Both alirocumab and evolocumab reduced MACE, stroke, and coronary revascularization. Evolocumab had a positive effect on myocardial infarction, but no effects were noted for alirocumab. In addition, alirocumab may not be as effective as evolocumab. NNT visualizes the magnitude of efficacy to assist in clinical decisions.

**Systematic review registration:**

[https://www.crd.york.ac.uk/PROSPERO/display_record.php?RecordID=344908], identifier [CRD42022344908].

## Introduction

Atherosclerotic cardiovascular disease (ASCVD) is a leading cause of death worldwide (1, 2). An elevated Low-Density Lipoprotein Cholesterol (LDL-C) level is an independent risk factor for ASCVD. Therefore, lowering LDL-C is a crucial strategy for both the primary and secondary prevention of ASCVD (3, 4). To achieve absolute or relative reductions in LDL-C among patients with high cardiovascular risk, statin lipid-lowering therapy is commonly recommended by current guidelines (5). However, a substantial proportion of patients who are either intolerant to or resistant to statins remain at significant residual risk for ASCVD events (6, 7). Therefore, many advocate the use of drug combinations to achieve greater efficacy and lower risk of cardiovascular events (8).

Anti-proprotein convertase subtilisin/Kexin type 9 (PCSK9) monoclonal antibodies are newer effective lipid-lowering drugs (9). The mechanisms of action of PCSK9 inhibitors: hepatocyte surface low-density lipoprotein receptor (LDLR) which is in charge of cellular uptake and subsequent LDL-C degradation is critical for cholesterol homeostasis (10). By interacting with LDLR on the hepatocyte surface, PCSK9 reduces the ability of LDLR to recycle. PCSK9 inhibitors can increase the density of LDLR on the cell surface and remove serum LDL particles for lipid control by preventing the interaction between circulating PCSK9 and LDLR (Supplementary Figure 1). Alirocumab and evolocumab, which are the representative drug of the PCSK9 inhibitors, have received approval from both the US Food and Drug Administration (FDA) and the European Medicine Agency (EMA). They are lipid-lowering injectable drugs demonstrated to reduce cardiovascular risk (11). They have been used for heterozygous familial hypercholesterolemia (HeFH) or clinical ASCVD requiring further lowering of LDL-C in addition to diet and maximally tolerated statin therapy (12–15). A growing body of literature recognizes the importance of PCSK9 inhibitors in reducing the occurrence of cardiovascular outcomes (16). Nevertheless, the independent report of relative indicators such as RR has not considered baseline risk, which may lead to underlying absolute risks being concealed and overestimation of results (17). Therefore, this study aims to assess the effect of alirocumab and evolocumab on all-cause mortality and major cardiovascular events considering the number needed to treat (NNT).

Number needed to treat as a commonly used absolute risk estimate is arguably the most clinically intuitive indicator (18). NNT has the advantage of being an easily interpretable summary of different treatment effects (19). NNT has both statistical and clinical meanings since it could transform an abstract rate into a specific frequency and translates clinical test results into clinical practice indicators. NNT also has the potential to support benefit-risk analysis and aid in the decision-making process for drug regulators (20, 21). NNT improves the objectivity, transparency, and repeatability of benefit-risk assessments since it can be used as a metric to quantitatively analyze the benefits and harms of medicines (18). In the reporting of clinical trials and other biomedical studies, NNT has been used frequently (22). In prior studies about ASCVD, only the NNT of statins and aspirin has been described (23, 24). Therefore, we explore the effectiveness of alirocumab and evolocumab in patients with established ASCVD utilizing NNT.

## Materials and methods

The protocol for this study has been registered in the International Systematic Prospective Register (PROSPERO, CRD42022344908).

### Research strategy and selection criteria

We systematically reviewed the literature according to the PRISMA (Preferred Reporting Items for Systematic Reviews and Meta-analyses) guidelines (25). We searched electronic databases of PubMed/Medline, Embase, CENTRAL (Cochrane Central Register of Controlled trials), and Web of Science up to May 23, 2022. The relevant keywords for searching included evolocumab, alirocumab, AMG145, Praluent, SAR236553, REGN727, and Repatha. The search strategy was presented in the Supplementary materials (Supplementary Text 1).

The inclusion criteria were: (1) Phase 2 or 3 randomized controlled trials (RCTs) comparing PCSK9 inhibitors to placebo or standards of care. (2) Patients with dyslipidemia and/or established ASCVD. (3) With follow-up duration of longer than one year, and (4) Trials reported the primary efficacy outcomes of interest: the composite endpoint of major adverse cardiovascular events (MACE), which were defined as cardiovascular death, myocardial infarction (MI), stroke, and coronary revascularization when available. No relevant outcomes were reported, meta-analyses, studies with duplicate data, and the number of participants who were less than 100 were excluded.

The screening strategy was primary searching through titles and abstracts and screening the remaining articles at the full-text level if any record fulfilled the study inclusion criteria.

### Data extraction

Two investigators (HW and YM) independently collected data with the pre-specified data collection forms and settled any discrepancies by discussion and consensus with the third reviewer (XX). Information collected from each study included the name of a registry, year of publication, sample size, type of medication, comorbidities, mean age, sex, duration of follow-up, baseline LDL-C, and efficacy outcomes. The primary outcome is MACE. Secondary outcomes included all-cause mortality, cardiovascular death, MI, stroke, and coronary revascularization. Two investigators (HW and YM) reviewed the studies and judged the risk of bias as low, unclear, or high risk in six different domains consisting of random sequence generation, allocation concealment, blinding of participants and personnel, blinding of outcome assessment, incomplete outcome data, selective reporting, and other sources of bias using the Cochrane Risk of Bias Tool (Supplementary Figure 2).

### Statistical analyses

We used the random effect model to obtain pooled RR and NNTs. Fixed effect models for each efficacy outcome were also given in the Supplementary. Heterogeneity was assessed using the χ*^2^* text, with *I*^2^ < 25%, 25% to 50%, and > 50% considered minimal, moderate, and substantial. We calculated NNT using the formula: NNT = 1/([1-RR] × CER), CER: the control (placebo) event rate. This study of 18 pooled CERs was obtained (Supplementary Table 1). NNT rounded up to the whole number for data interpretation. We calculated NNT together with its 95% CI. For rational interpretation of NNTs, the number needed to treat to benefit (NNTB) and the number needed to treat to harm (NNTH) are defined as the number of patients needed to be treated to reduce the number of outcomes by one and to increase the number of outcomes by one, respectively. If the 95% CI of NNTs crosses positive infinity, then it means there is no statistically significant. Due to the variations in trial duration, NNT may be somewhat biased when comparing alirocumab and evolocumab. Therefore, we used the method proposed by Laupacis et al. to adjust the length of follow-up (26). The formula: NNT: T × T ÷ S = NNT:S, NNT: T: the actual observed NNT, NNT:S: the adjusted NNT, T: the follow-up time, and S: the mean follow-up time. This method assumes that both the incidence of events and the treatment’s effect are constant over time.

Furthermore, we assumed that the MACE might be related to the mean age, published year, the mean follow-up duration, the percentage of male participants, diabetes mellitus, coronary artery disease, and taking stains. We used a random-effects univariate meta-regression to test this assumption. A 2-tailed *P* value < 0.05 was considered statistically significant.

All analyses were conducted using Review Manager V.5.4.1 (RevMan), R software, V.4.2.1, and Stata, V.17.0 (Stata Corp.).

## Results

### Included studies

**FIGURE 1:**
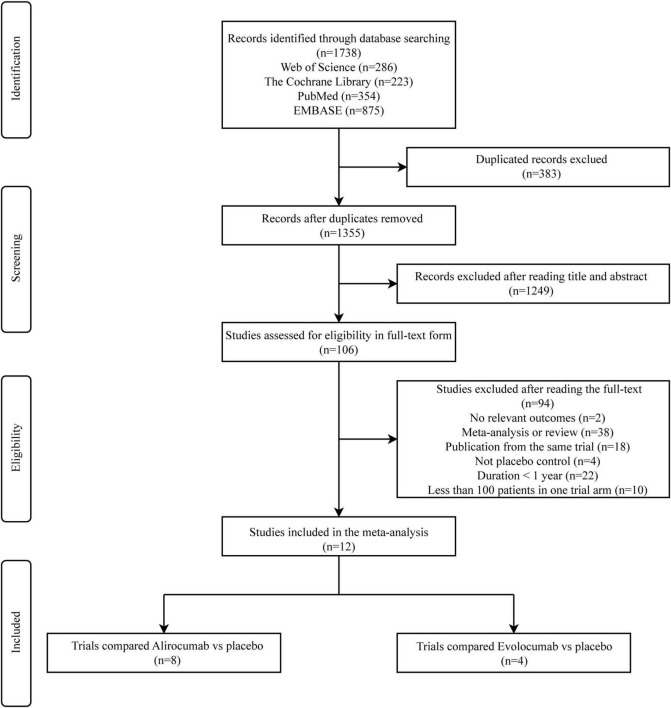
Flowchart of study selection for meta-analysis.

Figure 1 shows the flow diagram for study selection. (27–37). 1,738 records were identified by literature search (Web of Science 286 articles, the Cochrane Library 223 articles, Pubmed 354 articles, and EMBASE 875 articles). Finally, a total of 12 RCTs were identified which comprised 53,486 patients. At baseline, 27,674 patients were being treated with a PCSK9 inhibitor (12,071 with alirocumab and 15,603 with evolocumab), and 25,812 patients were being treated with placebo or standards of care. The mean follow-up time was 1.56 years. Table 1 presented the characteristics of included studies.

**TABLE 1:** Characteristics of interventions and populations at baseline included RCT.

Study	Year	n	DM, %	CAD, %	Drugs		Background treatment		Baseline characteristics					
							
					Treatment	Control	Statin, %	Ezetimibe, %	Mean Age, y	Men, %	Follow-up, y	Hypertension, %	BMI (kg/m^2^)	LDL-C at baseline (mg/dL)
ODYSSEY COMBO I NCT01644175	2015	314	43	78.2	Alirocumab	Placebo	100	8.2	63	65.8	1	NP	32.3	Alirocumab: 100.2
ODYSSEY LONG TERM NCT01507831	2015	2338	34.6	68.9	Alirocumab	Placebo	100	14.3	60	62.2	1.5	NP	30.4	Alirocumab: 122.7
ODYSSEY FH I NCT01623115	2015	485	9.1	42.7	Alirocumab	Placebo	100	57.2	52.6	55.1	1.5	39.6	28.8	Alirocumab: 144.7
ODYSSEY FH II NCT01709500	2015	248	9.1	42.7	Alirocumab	Placebo	100	66.3	52.6	55.1	1.5	39.6	28.8	Alirocumab: 134.6
ODYSSEY HIGH FH NCT01617655	2016	107	14.8	53	Alirocumab	Placebo	100	24.3	51	53	1.5	57.8	28.9	Alirocumab: 196.3
ODYSSEY JAPAN NCT02017898	2016	215	68.5	18.5	Alirocumab	Placebo	100	NP	60.8	60.6	1	NP	25.5	Alirocumab: 143.1
ODYSSEY OUTCOME NCT01663402	2018	18924	28.8	100	Alirocumab	Placebo	100	2.9	58.6	74.8	4	64.7	NP	Alirocumab: 92.0
PACMAN - AMI NCT03067844	2022	300	10.3	100	Alirocumab	Placebo	12.3	0.3	58.5	81	1	43.3	27.8	Alirocumab: 154.8
DESCARTES NCT01516879	2014	919	11.5	15.1	Evolocumab	Placebo	87.7	21	56.3	47.7	1	48.6	30.2	Evolocumab: 104.2
OSLER-1 NCT01439880	2014	1104	9.9	19	Evolocumab	SOC	62.5	26.7	56.3	55.1	1	NP	NP	Evolocumab: 138.5
GLAGOV NCT01813422	2016	968	20	100	Evolocumab	Placebo	98.6	2.1	59.8	72.2	1.5	83	29.5	Evolocumab: 92.6
FOURIER NCT01764633	2017	27564	36.6	100	Evolocumab	Placebo	100	5.2	62.5	75.4	2.2	80.1	NP	Evolocumab: 92.0

DM, diabetes mellitus; CAD, coronary artery disease; SOC, standards of care; BMI, body mass index.

### Endpoints

#### Major adverse cardiovascular events

**FIGURE 2:**
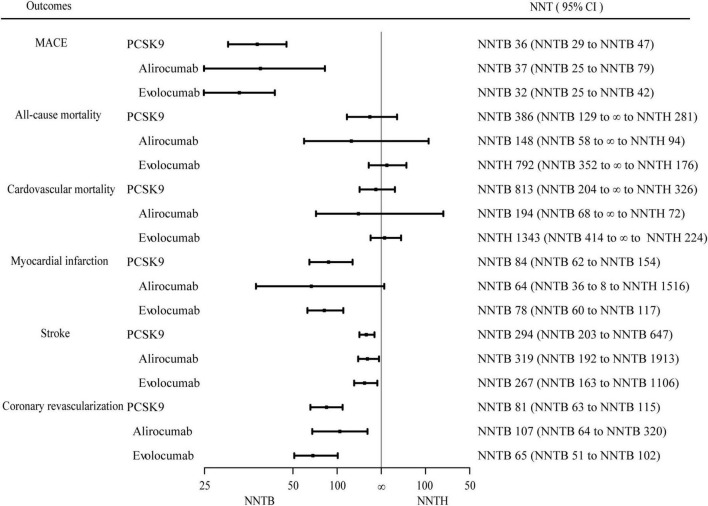
Efficacy endpoints for PCSK9 inhibitors vs. control. Effect of proprotein convertase subtilisin/Kexin type 9 (PCSK9) inhibitors on major adverse cardiovascular events, all-cause mortality, cardiovascular mortality, myocardial infarction, stroke, and coronary revascularization over 1.56 years. The number needed to treat (NNT) with the corresponding confidence intervals (CIs). MACE, major adverse cardiovascular events; NNT, the number needed to treat; NNTB, the number needed to treat to benefit; NNTH, the number needed to treat to harm.

Figure 2 presented the random-effects meta-analysis of the primary outcome. There were 53,486 patients with established ASCVD. The effect of PCSK9 inhibitors on MACE was statistically significant (RR 0.83, 95% CI 0.79–0.87) (Supplementary Figure 3), and the corresponding NNT was 36 (NNTB 29 to NNTB 47). When alirocumab and evolocumab analyses were conducted independently, they both reduced the incidence of MACE (RR 0.85, 95% CI 0.78–0.93 and RR 0.80, 95% CI 0.75–0.85, respectively). The NNT of MACE in ASCVD patients with alirocumab was 37 (NNTB 25 to NNTB 79), and evolocumab was 32 (NNTB 25 to NNTB 42).

**FIGURE 3:**
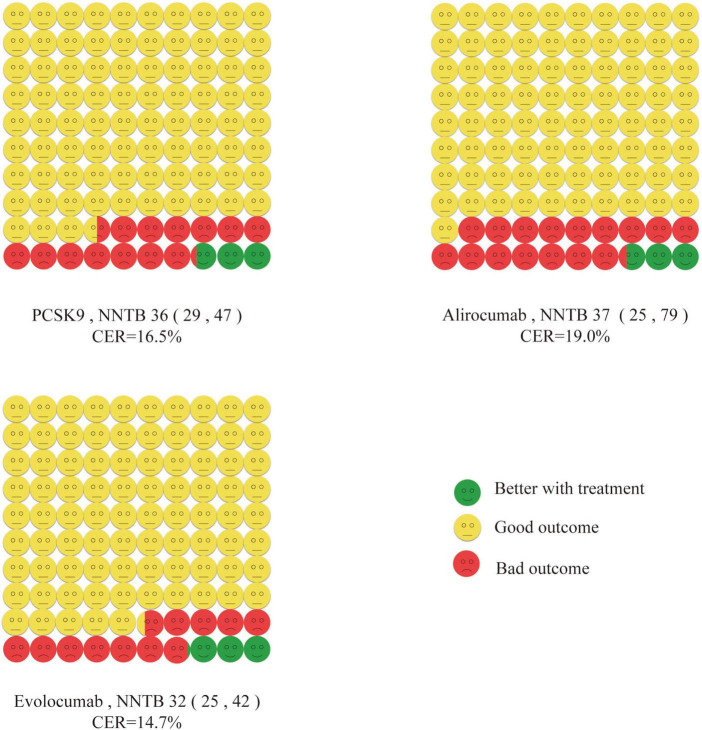
Cates plot. The effect of PCSK9 inhibitors in major adverse cardiovascular events is shown. 100 smiley faces represent 1,000 participants treated with PCSK9. Green face means no major adverse cardiovascular events if treated with PCSK9. Yellow face means no major adverse cardiovascular events even if not treated with PCSK9. Red face means major adverse cardiovascular events will occur even if treated with PCSK9.

The effect of PCSK9 inhibitors in MACE is shown in Figure 3. Direct comparison is not reasonable due to the difference in CER. For the convenience of comparison, NNT was transformed from the number of people required to treat to prevent one adverse event to the number of adverse events that could be prevented when treating 1,000 people for 1.56 years. The meanings of these two indicators are the same. A total of 12 studies with CER of 16.5% were included, and the treatment of 1,000 ASCVD patients by PCSK9 inhibitors would benefit an average benefit of 27-28 individuals. Alirocumab was included in eight studies with CER of 19% and benefited an average of 27-28 people treating 1,000 patients with ASCVD. Evolocumab was included in 4 studies with CER of 14.7% and benefited an average of 31-32 people treating 1,000 patients with ASCVD.

#### All-cause mortality

All trials evaluated the data of PCSK9 inhibitors on all-cause mortality. Compared with no treatment with PCSK9 inhibitors, PCSK9 inhibitors were not associated with a statistically significant change in all-cause mortality (RR 0.92, 95% CI 0.76–1.11) (Supplementary Figure 4) with a value of 386 (NNTB 129 to ∞ to NNTH 281). Alirocumab and evolocumab were not statistically significant in all-cause mortality (*P* = 0.38 and *P* = 0.59, respectively).

#### Cardiovascular mortality

Cardiovascular mortality analysis included eleven RCTs (52,508 patients). The NNT of cardiovascular mortality was 813 (NNTB 204 to ∞ to NNTH 326). Between PCSK9 inhibitors and controls, there were no significant differences in cardiovascular mortality (RR 0.94, 95% CI 0.76–1.15) (Supplementary Figure 5). The effect of alirocumab and evolocumab on cardiovascular mortality was also statistically non-significant (*P* = 0.50 and *P* = 0.65, respectively).

#### Myocardial infarction

All trials reported the data of PCSK9 inhibitors therapy on MI. The effect of PCSK9 inhibitors on MI was statistically significant (RR 0.78, 95% CI 0.70–0.88) (Supplementary Figure 6). The NNT of PCSK9 inhibitors on MI was 84 (95% CI, NNTB 62 to NNTB 154). When alirocumab and evolocumab were performed separately, evolocumab reduced MI (RR 0.73, 95% CI 0.65–0.82), and NNT was 78 (NNTB 60 to NNTB 117). Alirocumab had no effect (*P* = 0.06).

#### Stroke

There were 10 trials (52,267 patients) that reported data on stroke. Compared with no treatment with PCSK9 inhibitors, the efficacy of PCSK9 inhibitors on stroke was statistically significant (RR 0.78, 95% CI 0.68–0.90) (Supplementary Figure 7), and NNT was 294 (NNTB 203 to NNTB 647). When alirocumab and evolocumab were performed separately, they both lowered the occurrence of heart failure (RR 0.76, 95% CI 0.60–0.96 and RR 0.79, 95% CI 0.66–0.95, respectively). The NNT of stroke in patients with alirocumab was 319 (NNTB 192 to NNTB 1913), and evolocumab was 267 (NNTB 163 to NNTB 1106).

#### Coronary revascularization

Coronary revascularization was evaluated in 11 trials (52,567 patients). Compared with the control group, treatment with PCSK9 inhibitors was associated with a statistically significant reduction in coronary revascularization (RR 0.83, 95% CI 0.78–0.88) (Supplementary Figure 8). The NNT of coronary revascularization was 81 (95% CI, NNTB 63 to NNTB 115). When alirocumab and evolocumab were performed separately, they reduced coronary revascularization incidence (RR 0.88, 95% CI 0.80–0.96 and RR 0.78, 95% CI 0.72–0.86, respectively). The corresponding NNT for alirocumab was 107 (NNTB 64 to NNTB 320), and the NNT for evolocumab was 65 (NNTB 51 to NNTB 102).

#### Meta-regression analysis and publication bias

The purpose of conducting meta-regression is to evaluate the magnitude and sources of heterogeneity among studies. According to predefined baseline characteristics, there are many trial subgroups: mean age, published year, the percentage of male participants, diabetes mellitus, coronary artery disease or statin, and follow-up years. We further identified study heterogeneity by focusing on the relationship between clinical characteristics and intervention effect sizes. There was no evidence of differences in the effects of PCSK9 inhibitors on MACE among trial subgroups (all *P* > 0.05 for heterogeneity) (Supplementary Table 2). There was no evidence for publication bias in the funnel plots (Supplementary Figure 9), as Begg’s rank correlations (*P* = 0.15) and Egger’s linear regression (*P* = 0.80) have been proven with statistics (Supplementary Figure 10).

## Discussion

To the best of our knowledge, this study is the first meta-analysis focusing on NNT and assessing the efficacy of PCSK9 inhibitors on cardiovascular events in patients with ASCVD. This study determines the effectiveness of PCSK9 inhibitors to prevent MACE among patients with established ASCVD by calculating NNTs.

Both alirocumab and evolocumab could prevent MACE, stroke, and coronary revascularization. Prior studies have noted the importance of PCSK9 inhibitors to reduce the risk of MACE. PCSK9 inhibitors increase the availability of cell surface LDL receptors and thus reduce plasma LDL-C levels (38, 39). It provides most cardiovascular benefits by lowering LDL-C, but lipid effects alone are insufficient to fully explain the changes in clinical cardiovascular event rates. The non-lipidic effects of PCSK9 inhibitors also play a role in this process to some extent. Firstly, PCSK9 inhibitors can interfere with the atherosclerotic inflammatory response to a certain extent and further achieve vascular benefit. Secondly, coagulation status is also an influential factor in acute coronary syndrome. It was found that PCSK9 can directly activate platelets on the one hand, and on the other hand, it can also lead to abnormal lipid and inflammatory status and thus indirectly lead to a hypercoagulable state of the body (10). PCSK9 inhibitors significantly inhibit platelet hyperactivation by neutralizing PCSK to reduce the incidence of cardiovascular events. PCSK9 inhibitors are expected to change the landscape of LDL-lowering therapy in future clinical drug therapy (7, 40).

This study reported adding PCSK9 inhibitors to statins may reduce the risk of MI and stroke but does not affect mortality. The insignificant effect on mortality was most likely due to the short follow-up period. Several studies have found that PCSK9 inhibitors over time enhanced the beneficial cardiovascular effects (32, 37, 41). Although studies with at least a year of follow-up were included in the current meta-analysis, the median follow-up time for all trials was only 1.56 years. More long-term follow-up studies are needed (39). The similar finding on mortality was also reported by Safi U Khan et al. (10, 16). However, the used RRs alone do not reflect the magnitude of baseline risk. RRs convey relative differences not absolute differences in the outcomes (42). It is interpretable only if the occurrence rate in the control group is also mentioned (43). For example, the RRs for MACE and coronary revascularization in our report were approximately the same, however, the NNT is very different. Considering the baseline data, we used the relative effect indicator RRs in our study and added the absolute effect indicator NNT for evaluation. NNT may be useful in assisting doctors to understand the specific risks and advantages of treatment strategies (44).

Another finding indicates that evolocumab is more effective than alirocumab. Among patients with ASCVD, only evolocumab but not alirocumab could reduce MI. Several previous studies have also concluded that alirocumab had a weaker LDL-C-lowering ability than evolocumab (45–47). NNT conveys the absolute size of differences in outcomes between treatments in a readily interpretable way. For example, both alirocumab and evolocumab may reduce the incidence of stroke. If clinicians just focus solely on RRs, without considering patient personality characteristics and drug differences when making drug selections, they will probably choose alirocumab due to its lower RRs. However, when we consider the risk at baseline, we observed that the NNT for alirocumab is 319, while the NNT for evolocumab is 267, which means evolocumab performed better in treating the same number of patients than alirocumab. NNT does not only have the advantage of showing true efficacy but is also more convincing in the interpretation of conclusions than RRs (48, 49). However, due to the lack of direct face-to-face studies, this conclusion needs to be further investigated.

Increasingly, top medical journals require reporting of NNT as a supplemental indicator (50). NNT has advantages over RR by expressing efficacy by combining baseline risk and treated risk reduction. The NNT is more useful than an absolute risk because it tells clinicians and patients more specifically how much effort they must exert to prevent an event. Previous studies have also used an absolute effect metric, incidence per 1,000 persons over five years, for assessment. Yet this is not consistent with many included trials in our study. Five years far exceeds the true experimental follow-up time. It is unrealistic to assume that the five-year baseline risk is constant. The advantage of NNT over incidence per 1,000 persons over five years is a more accurate assessment of baseline data, enabling more realistic and credible conclusions.

This study also aids with the cost-effectiveness analysis of drugs (51–53). When two PCSK9 inhibitors became commercially available in 2015, they did improve the clinical prognosis for secondary prevention, but the annual retail cost was nearly $14,000. Expensive market prices are a significant barrier to patient access (54–56). When we need to conduct a cost-effectiveness analysis, the most frequently advised numerical metric for healthcare professionals is NNT (57).

There are several limitations of our analysis when interpreting the results. Firstly, baseline risk and follow-up time have a significant influence to calculate NNT, but they varied for each included study. To address this variation, we pooled the baseline risks and the mean follow-up time of the trials involved in the study to calculate an approximation of the true value. Secondly, this study primarily analyzed all-cause mortality and major cardiovascular events and excluded safety outcomes. Future studies could investigate this further. Finally, this meta-analysis was performed at study level data rather than the patient level. Therefore, we could only explore meta-regressions of the association between baseline levels and outcomes but not conduct subgroup analyses between some potential factors. We attempted to contact the authors to obtain individual data from their trials and hope to report these data in future trials.

## Conclusion

In conclusion, this study suggests that preventing one patient from MACE needed to treat 36 patients with ASCVD with PCSK9 inhibitors for 1.56 years. Both alirocumab and evolocumab, which are currently in clinical use, have reduced MACE, including stroke and coronary revascularization in patients with ASCVD. Evolocumab has also shown promising results in MI. In addition, alirocumab may not be as effective as evolocumab. NNTs visualize the magnitude of efficacy. These findings provide important and useful guidance for clinicians in treating and managing patients with ASCVD.

## Data availability statement

The original contributions presented in this study are included in the article/Supplementary material, further inquiries can be directed to the corresponding author.

## Author contributions

H-FW: investigation, validation, visualization, formal analysis, and manuscript drafting. Y-CM: investigation, validation, visualization, and formal analysis. X-YX: formal analysis, writing—review, and editing. S-YZ, D-DH, S-YG, KS, and CG: investigation. Q-BT: supervision, conceptualization, and methodology. All authors contributed to manuscript revision, read, and approved the submitted version.
