# Supplementary material for: Effect of alirocumab and evolocumab on all-cause mortality and major cardiovascular events: A meta-analysis focusing on the number needed to treat
Source: Front Cardiovasc Med. 2022 Dec 2;9:1016802. doi: 10.3389/fcvm.2022.1016802 (PMC9755489; doi:10.3389/fcvm.2022.1016802)
Supplement: Supplementary file 1 [file Data_Sheet_1.PDF]

Supplementary Material

1 Supplementary Text 1. Search strategies

Pubmed n=354

1, "Dyslipidemias"[MeSH Terms] OR "Dyslipidemia"[Title/Abstract] OR "Dyslipoproteinemias"[Title/Abstract] OR "Dyslipoproteinemia"[Title/Abstract] OR "Atherosclerosis"[MeSH Terms] OR "Atheroscleroses"[Title/Abstract] OR "Atherogenesis"[Title/Abstract]

2,"alirocumab"[Supplementary Concept] OR "SAR236553"[Title/Abstract] OR "SAR-236553"[Title/Abstract] OR "REGN-727"[Title/Abstract] OR (("antibodies, monoclonal"[MeSH Terms] OR ("antibodies"[All Fields] AND "monoclonal"[All Fields]) OR "monoclonal antibodies"[All Fields] OR ("monoclonal"[All Fields] AND "antibody"[All Fields]) OR "monoclonal antibody"[All Fields]) AND "REGN727"[Title/Abstract]) OR "regn727 monoclonal antibody"[Title/Abstract] OR "REGN727"[Title/Abstract] OR "praluent"[Title/Abstract] OR "evolocumab"[Supplementary Concept] OR "Repatha"[Title/Abstract] OR "AMG-145"[Title/Abstract] OR "AMG-145"[Title/Abstract]

3, "randomized controlled trial"[Publication Type] OR "controlled clinical trial"[Title/Abstract] OR "random allocation"[Title/Abstract] OR "double-blind"[Title/Abstract] OR "single blind"[Title/Abstract] OR "Placebo"[Title/Abstract] OR "Randomly"[Title/Abstract] OR "Randomized"[Title/Abstract] OR "clinical trial*"[Title/Abstract] OR "trial*"[Title/Abstract] OR "RCT"[Title/Abstract] OR "random*"[Title/Abstract]

4, #1 AND #2 AND #3

WOS n=286

1, TS=（Dyslipidaemia OR Dyslipidemia OR Dyslipoproteinemias OR Dyslipoproteinemia OR Atherosclerosis OR Atheroscleroses OR Atherogenesis）

2, TS=(alirocumab OR SAR236553 OR SAR-236553 OR REGN-727 OR monoclonal antibody REGN727 OR REGN727 monoclonal antibody OR REGN727 OR praluent OR evolocumab OR Repatha OR AMG-145 OR AMG 145)

3, TS=(Randomized Controlled Trial OR controlled clinical trial OR random allocation OR double-blind OR single- blind OR Placebo OR Randomly OR randomized OR clinical trial* OR trial* OR RCT OR Random*)

4, #1 AND #2 AND #3

Cochrane Library n=223

1, (Dyslipidaemia OR Dyslipidemia OR Dyslipoproteinemias OR Dyslipoproteinemia OR Atherosclerosis OR Atheroscleroses OR Atherogenesis) :ti,ab,kw

2, (alirocumab OR SAR236553 OR SAR-236553 OR REGN-727 OR monoclonal antibody REGN727 OR REGN727 monoclonal antibody OR REGN727 OR praluent OR evolocumab OR Repatha OR AMG-145 OR AMG 145):ti,ab,kw

3, (Randomized Controlled Trial OR controlled clinical trial OR random allocation OR double-blind OR Placebo OR Randomly OR randomized OR clinical trial* OR trial* OR RCT OR Random*):ti,ab,kw

4, #1 AND #2 AND #3

Embase n=875

1, 'dyslipidaemia'/exp OR 'dyslipidaemia' OR 'Dyslipidemia':ab,ti OR 'Dyslipoproteinemias':ab,ti OR 'Dyslipoproteinemia':ab,ti OR 'Atherosclerosis'/exp OR ' Atherosclerosis' OR 'Atheroscleroses':ab,ti OR 'Atherogenesis':ab,ti

2, 'alirocumab'/exp OR 'alirocumab' OR 'sar236553':ab,ti OR 'sar-236553':ab,ti OR 'regn-727':ab,ti OR 'monoclonal antibody regn727':ab,ti OR 'regn727 monoclonal antibody':ab,ti OR 'regn727':ab,ti OR 'praluent':ab,ti OR 'evolocumab'/exp OR evolocumab OR 'repatha':ab,ti OR 'amg-145':ab,ti OR 'amg 145':ab,ti

3, 'Randomized Controlled Trial'/exp OR 'Randomized Controlled Trial' OR 'controlled clinical trial':ab,ti OR 'random allocation':ab,ti OR 'double-blind':ab,ti OR 'single-blind':ab,ti OR 'Placebo':ab,ti OR 'Randomly':ab,ti OR 'randomized':ab,ti OR 'clinical trial*':ab,ti OR 'trial*':ab,ti OR 'RCT':ab,ti OR 'Random*':ab,ti

4, #1 AND #2 AND #3

Supplementary Table 1. The pooled CERs of PCSK9 inhibitors.

Outcomes	Drug type	CER (control event rate)
major vascular events	PCSK9 inhibitors	0.165
	alirocumab	0.190
	evolocumab	0.147
all-cause mortality	PCSK9 inhibitors	0.032
	alirocumab	0.037
	evolocumab	0.029
cardiovascular death	PCSK9 inhibitors	0.021
	alirocumab	0.026
	evolocumab	0.017
myocardial infarction	PCSK9 inhibitors	0.054
	alirocumab	0.069
	evolocumab	0.044
stroke	PCSK9 inhibitors	0.017
	alirocumab	0.014
	evolocumab	0.018
coronary revascularization	PCSK9 inhibitors	0.075
	alirocumab	0.081
	evolocumab	0.071

3 Supplementary Table 2. Univariate meta-regression analysis of PCSK9 inhibitors on major vascular event

Model	Covariate	Classification	No. of Studies	*P* Value for Heterogeneity
1	Type of PCSK9 inhibitors	Alirocumab	8	0.221
		Evolocumab	4	
2	Mean age, y	< 60	8	0.419
		⩾60	4	
3	Published year		14	0.274
4	Men, %		14	0.219
5	Follow-up, y		14	0.380
6	DM, %		14	0.333
7	CAD, %		14	0.293
8	statins, %		14	0.546

DM, Diabetes mellitus; CAD, coronary artery disease

4 **Supplementary Figure 1.**

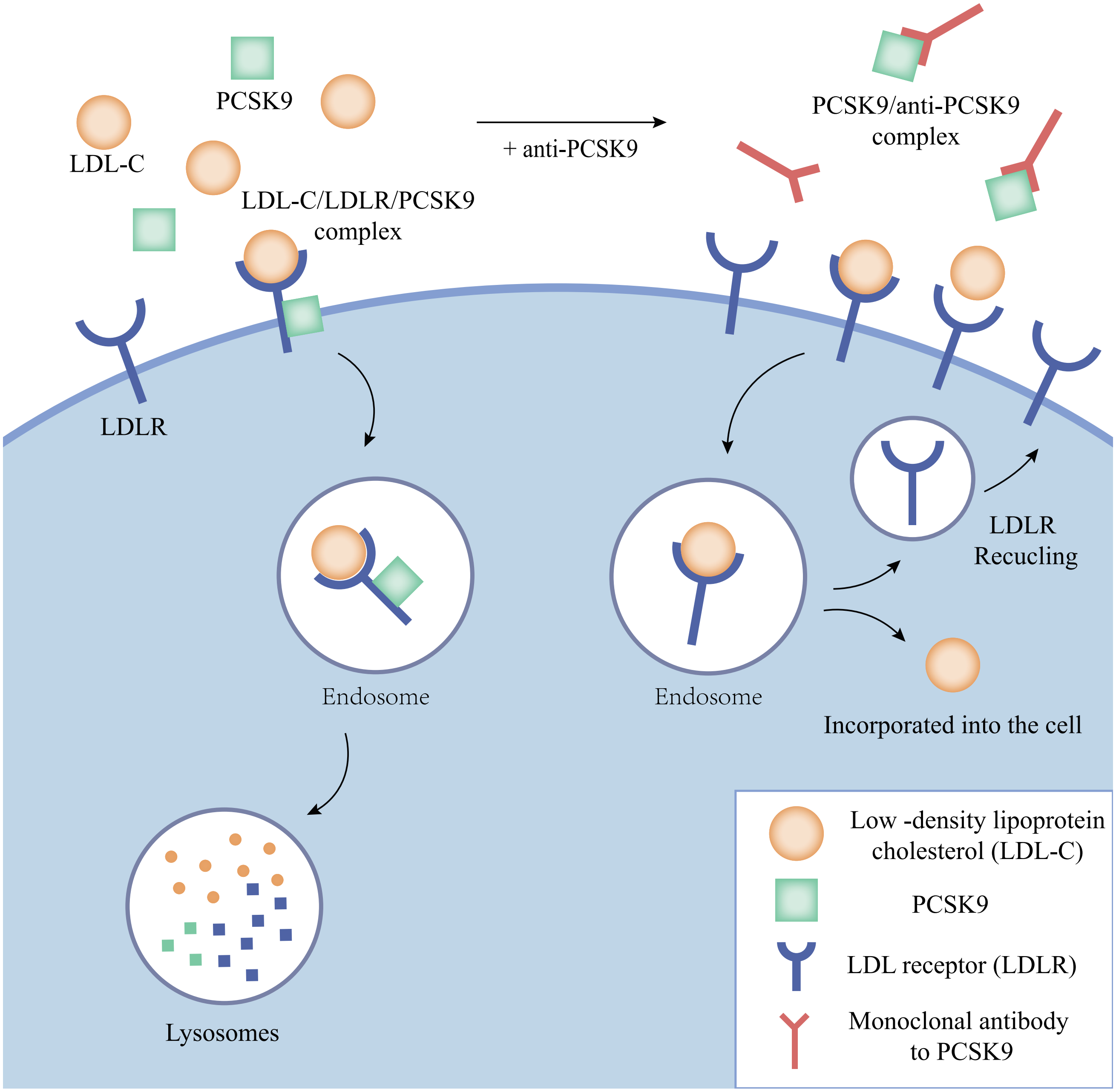

Supplementary Figure 1. The mechanisms of action of PCSK9 inhibitors.

5 Supplementary Figure 2.

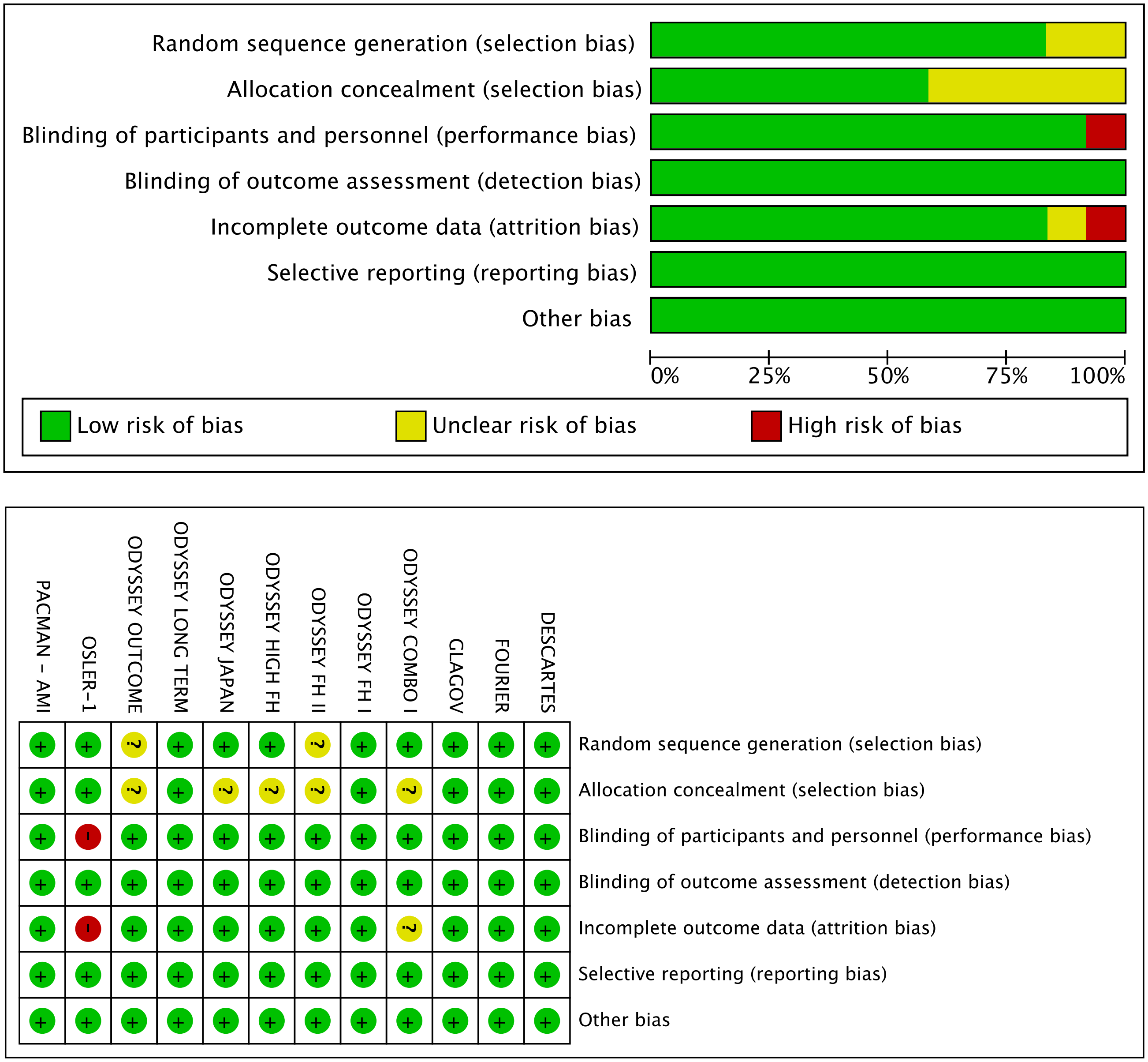

Supplementary Figure 2. Results of risk assessment.

6 Supplementary Figure 3.

(A)

Study or Subgroup	Antibody Events	Antibody Total	No antibody Events	No antibody Total	Weight	Risk Ratio M-H, Random, 95% CI
**Alirocumab**						
ODYSSEY COMBO I	7	207	3	107	0.1%	1.21 [0.32, 4.57]
ODYSSEY FH I	7	322	3	163	0.1%	1.18 [0.31, 4.51]
ODYSSEY FH II	2	167	2	81	0.1%	0.49 [0.07, 3.38]
ODYSSEY HIGH FH	9	72	0	35	0.0%	9.37 [0.56, 156.52]
ODYSSEY JAPAN	3	143	2	72	0.1%	0.76 [0.13, 4.42]
ODYSSEY LONG TERM	75	1550	51	788	2.0%	0.75 [0.53, 1.06]
ODYSSEY OUTCOME	1708	9462	1973	9462	47.7%	0.87 [0.82, 0.92]
PACMAN - AMI	16	148	31	152	0.8%	0.53 [0.30, 0.93]
**Subtotal (95% CI)**		**12071**		**10860**	**50.9%**	**0.85 [0.78, 0.93]**
Total events	1827		2065			
Heterogeneity: $Tau^2 = 0.00$; $Chi^2 = 7.14$, df = 7 (P = 0.41); $I^2 = 2\%$						
Test for overall effect: Z = 3.58 (P = 0.0003)						
**Evolocumab**						
DESCARTES	3	599	0	320	0.0%	3.75 [0.19, 72.28]
FOURIER	1685	13784	2106	13780	46.2%	0.80 [0.75, 0.85]
GLAGOV	62	484	83	484	2.6%	0.75 [0.55, 1.01]
OSLER-1	13	736	7	368	0.3%	0.93 [0.37, 2.31]
**Subtotal (95% CI)**		**15603**		**14952**	**49.1%**	**0.80 [0.75, 0.85]**
Total events	1763		2196			
Heterogeneity: $Tau^2 = 0.00$; $Chi^2 = 1.34$, df = 3 (P = 0.72); $I^2 = 0\%$						
Test for overall effect: Z = 7.55 (P < 0.00001)						
**Total (95% CI)**		**27674**		**25812**	**100.0%**	**0.83 [0.79, 0.87]**
Total events	3590		4261			
Heterogeneity: $Tau^2 = 0.00$; $Chi^2 = 11.55$, df = 11 (P = 0.40); $I^2 = 5\%$						
Test for overall effect: Z = 7.56 (P < 0.00001)						
Test for subgroup differences: $Chi^2 = 1.34$, df = 1 (P = 0.25), $I^2 = 25.1\%$						

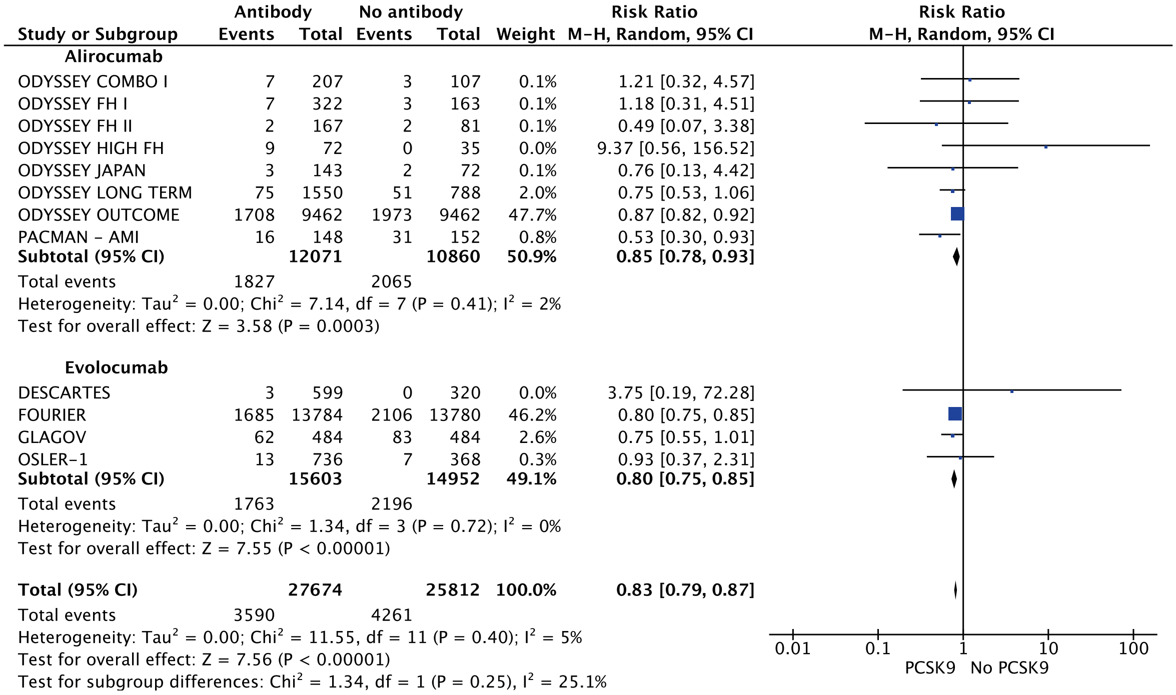

(B)

Study or Subgroup	Antibody Events	Antibody Total	No antibody Events	No antibody Total	Weight	Risk Ratio M-H, Fixed, 95% CI
**Alirocumab**						
ODYSSEY COMBO I	7	207	3	107	0.1%	1.21 [0.32, 4.57]
ODYSSEY FH I	7	322	3	163	0.1%	1.18 [0.31, 4.51]
ODYSSEY FH II	2	167	2	81	0.1%	0.49 [0.07, 3.38]
ODYSSEY HIGH FH	9	72	0	35	0.0%	9.37 [0.56, 156.52]
ODYSSEY JAPAN	3	143	2	72	0.1%	0.76 [0.13, 4.42]
ODYSSEY LONG TERM	75	1550	51	788	1.6%	0.75 [0.53, 1.06]
ODYSSEY OUTCOME	1708	9462	1973	9462	46.1%	0.87 [0.82, 0.92]
PACMAN - AMI	16	148	31	152	0.7%	0.53 [0.30, 0.93]
**Subtotal (95% CI)**		**12071**		**10860**	**48.7%**	**0.86 [0.81, 0.91]**
Total events	1827		2065			
Heterogeneity: $Chi^2 = 7.14$, df = 7 (P = 0.41); $I^2 = 2\%$						
Test for overall effect: Z = 5.19 (P < 0.00001)						
**Evolocumab**						
DESCARTES	3	599	0	320	0.0%	3.75 [0.19, 72.28]
FOURIER	1685	13784	2106	13780	49.2%	0.80 [0.75, 0.85]
GLAGOV	62	484	83	484	1.9%	0.75 [0.55, 1.01]
OSLER-1	13	736	7	368	0.2%	0.93 [0.37, 2.31]
**Subtotal (95% CI)**		**15603**		**14952**	**51.3%**	**0.80 [0.75, 0.85]**
Total events	1763		2196			
Heterogeneity: $Chi^2 = 1.34$, df = 3 (P = 0.72); $I^2 = 0\%$						
Test for overall effect: Z = 7.53 (P < 0.00001)						
**Total (95% CI)**		**27674**		**25812**	**100.0%**	**0.83 [0.80, 0.86]**
Total events	3590		4261			
Heterogeneity: $Chi^2 = 11.55$, df = 11 (P = 0.40); $I^2 = 5\%$						
Test for overall effect: Z = 9.03 (P < 0.00001)						
Test for subgroup differences: $Chi^2 = 3.13$, df = 1 (P = 0.08), $I^2 = 68.1\%$						

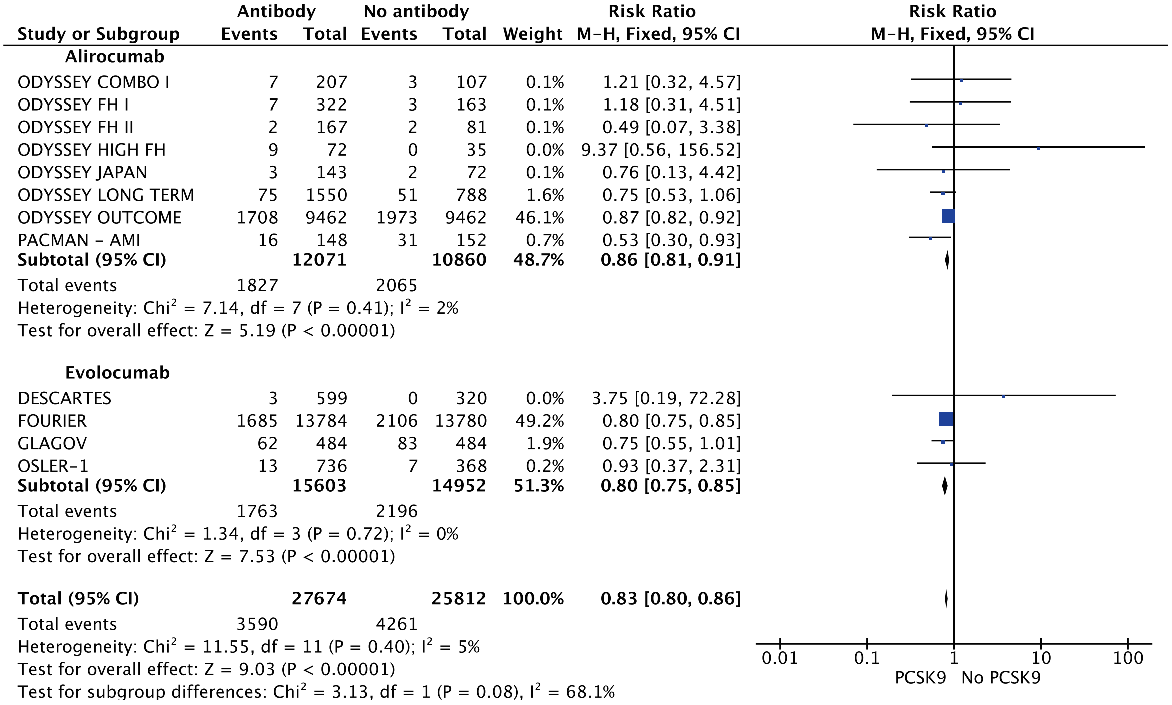

Supplementary Figure 3. Risk estimates using random effect (A) and fixed effect (B) models for major vascular events.

7 Supplementary Figure 4.

(A)

Study or Subgroup	Antibody Events	Antibody Total	No antibody Events	No antibody Total	Weight	Risk Ratio M-H, Random, 95% CI
**Alirocumab**						
ODYSSEY COMBO I	2	207	1	107	0.6%	1.03 [0.09, 11.27]
ODYSSEY FH I	6	322	0	163	0.4%	6.60 [0.37, 116.45]
ODYSSEY FH II	0	167	0	81		Not estimable
ODYSSEY HIGH FH	0	72	0	35		Not estimable
ODYSSEY JAPAN	0	143	0	72		Not estimable
ODYSSEY LONG TERM	8	1550	10	788	3.8%	0.41 [0.16, 1.03]
ODYSSEY OUTCOME	334	9462	392	9462	45.0%	0.85 [0.74, 0.98]
PACMAN - AMI	2	148	1	152	0.6%	2.05 [0.19, 22.41]
**Subtotal (95% CI)**		**12071**		**10860**	**50.4%**	**0.81 [0.51, 1.30]**
Total events	352		404			
Heterogeneity: $Tau^2 = 0.08$; $Chi^2 = 4.95$, df = 4 (P = 0.29); $I^2 = 19\%$						
Test for overall effect: Z = 0.87 (P = 0.38)						
**Evolocumab**						
DESCARTES	2	599	0	320	0.4%	2.67 [0.13, 55.55]
FOURIER	444	13784	426	13780	47.0%	1.04 [0.91, 1.19]
GLAGOV	3	484	4	484	1.5%	0.75 [0.17, 3.33]
OSLER-1	1	736	2	368	0.6%	0.25 [0.02, 2.75]
**Subtotal (95% CI)**		**15603**		**14952**	**49.6%**	**1.04 [0.91, 1.18]**
Total events	450		432			
Heterogeneity: $Tau^2 = 0.00$; $Chi^2 = 1.91$, df = 3 (P = 0.59); $I^2 = 0\%$						
Test for overall effect: Z = 0.54 (P = 0.59)						
**Total (95% CI)**		**27674**		**25812**	**100.0%**	**0.92 [0.76, 1.11]**
Total events	802		836			
Heterogeneity: $Tau^2 = 0.01$; $Chi^2 = 11.24$, df = 8 (P = 0.19); $I^2 = 29\%$						
Test for overall effect: Z = 0.88 (P = 0.38)						
Test for subgroup differences: $Chi^2 = 0.97$, df = 1 (P = 0.33), $I^2 = 0\%$						

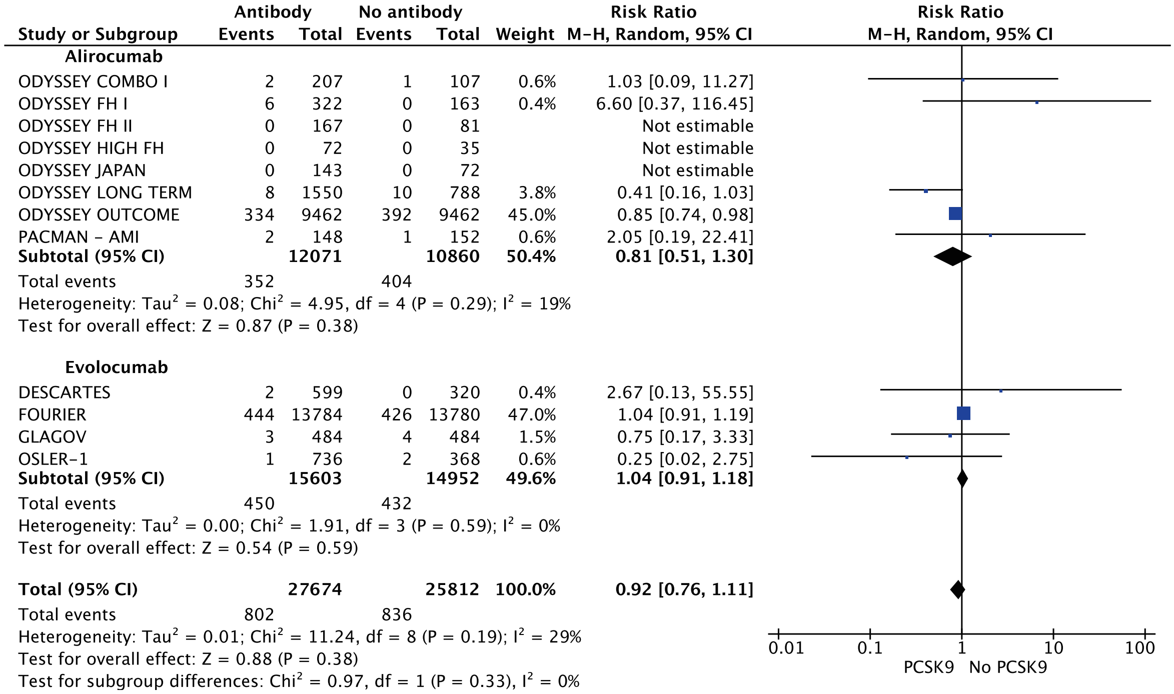

(B)

Study or Subgroup	Antibody Events	Antibody Total	No antibody Events	No antibody Total	Weight	Risk Ratio M-H, Fixed, 95% CI
**Alirocumab**						
ODYSSEY COMBO I	2	207	1	107	0.2%	1.03 [0.09, 11.27]
ODYSSEY FH I	6	322	0	163	0.1%	6.60 [0.37, 116.45]
ODYSSEY FH II	0	167	0	81		Not estimable
ODYSSEY HIGH FH	0	72	0	35		Not estimable
ODYSSEY JAPAN	0	143	0	72		Not estimable
ODYSSEY LONG TERM	8	1550	10	788	1.6%	0.41 [0.16, 1.03]
ODYSSEY OUTCOME	334	9462	392	9462	46.6%	0.85 [0.74, 0.98]
PACMAN - AMI	2	148	1	152	0.1%	2.05 [0.19, 22.41]
**Subtotal (95% CI)**		**12071**		**10860**	**48.5%**	**0.85 [0.74, 0.98]**
Total events	352		404			
Heterogeneity: $Chi^2 = 4.95$, df = 4 (P = 0.29); $I^2 = 19\%$						
Test for overall effect: Z = 2.26 (P = 0.02)						
**Evolocumab**						
DESCARTES	2	599	0	320	0.1%	2.67 [0.13, 55.55]
FOURIER	444	13784	426	13780	50.6%	1.04 [0.91, 1.19]
GLAGOV	3	484	4	484	0.5%	0.75 [0.17, 3.33]
OSLER-1	1	736	2	368	0.3%	0.25 [0.02, 2.75]
**Subtotal (95% CI)**		**15603**		**14952**	**51.5%**	**1.04 [0.91, 1.18]**
Total events	450		432			
Heterogeneity: $Chi^2 = 1.91$, df = 3 (P = 0.59); $I^2 = 0\%$						
Test for overall effect: Z = 0.55 (P = 0.59)						
**Total (95% CI)**		**27674**		**25812**	**100.0%**	**0.95 [0.86, 1.04]**
Total events	802		836			
Heterogeneity: $Chi^2 = 11.24$, df = 8 (P = 0.19); $I^2 = 29\%$						
Test for overall effect: Z = 1.13 (P = 0.26)						
Test for subgroup differences: $Chi^2 = 4.13$, df = 1 (P = 0.04), $I^2 = 75.8\%$						

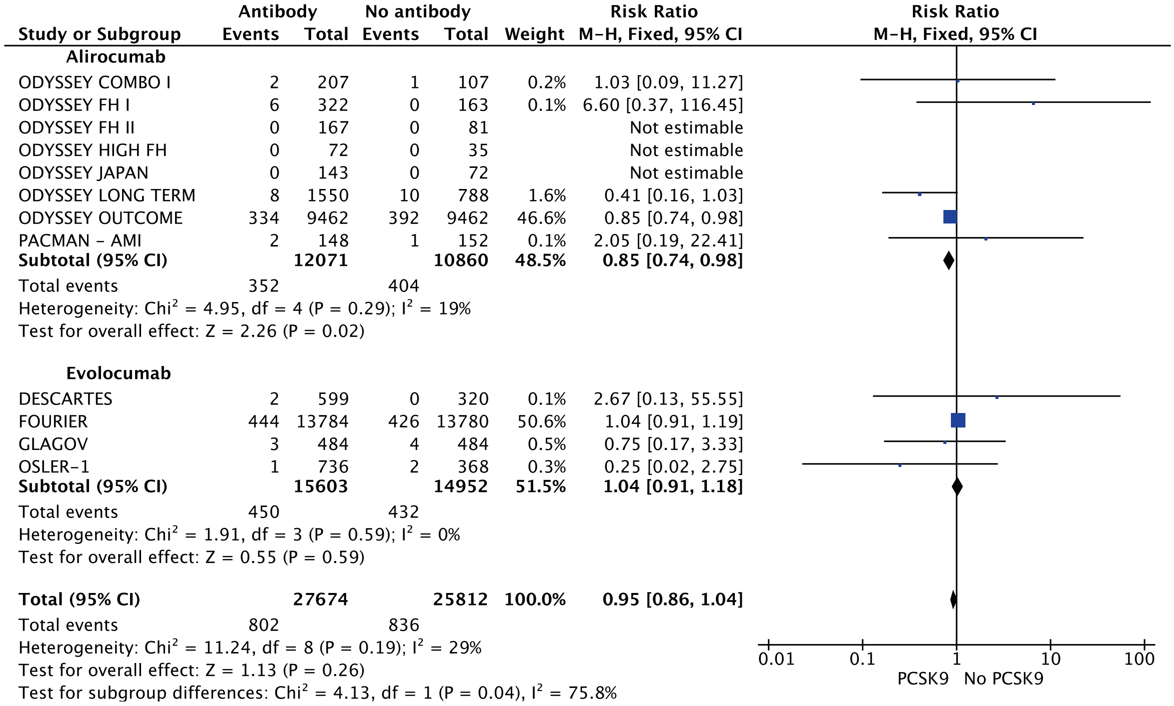

Supplementary Figure 4. Risk estimates using random effect (A) and fixed effect (B) models for all-cause death.

8 Supplementary Figure 5.

(A)

Study or Subgroup	Antibody Events	Antibody Total	No antibody Events	No antibody Total	Weight	Risk Ratio M-H, Random, 95% CI
**Alirocumab**						
ODYSSEY COMBO I	1	207	1	107	0.6%	0.52 [0.03, 8.18]
ODYSSEY FH I	3	322	0	163	0.5%	3.55 [0.18, 68.40]
ODYSSEY FH II	0	167	0	81		Not estimable
ODYSSEY HIGH FH	0	72	0	35		Not estimable
ODYSSEY JAPAN	0	143	0	72		Not estimable
ODYSSEY LONG TERM	4	1550	7	788	2.7%	0.29 [0.09, 0.99]
ODYSSEY OUTCOME	240	9462	271	9462	47.6%	0.89 [0.75, 1.05]
PACMAN – AMI	2	148	0	152	0.5%	5.13 [0.25, 106.05]
**Subtotal (95% CI)**		**12071**		**10860**	**51.9%**	**0.79 [0.40, 1.57]**
Total events	250		279			
Heterogeneity: $Tau^2 = 0.19$; $Chi^2 = 5.45$, df = 4 (P = 0.24); $I^2 = 27\%$						
Test for overall effect: Z = 0.67 (P = 0.50)						
**Evolocumab**						
DESCARTES	2	599	0	320	0.5%	2.67 [0.13, 55.55]
FOURIER	251	13784	240	13780	46.9%	1.05 [0.88, 1.25]
OSLER–1	1	726	2	368	0.7%	0.25 [0.02, 2.79]
**Subtotal (95% CI)**		**15109**		**14468**	**48.1%**	**1.04 [0.87, 1.24]**
Total events	254		242			
Heterogeneity: $Tau^2 = 0.00$; $Chi^2 = 1.71$, df = 2 (P = 0.43); $I^2 = 0\%$						
Test for overall effect: Z = 0.45 (P = 0.65)						
**Total (95% CI)**		**27180**		**25328**	**100.0%**	**0.94 [0.76, 1.15]**
Total events	504		521			
Heterogeneity: $Tau^2 = 0.02$; $Chi^2 = 9.14$, df = 7 (P = 0.24); $I^2 = 23\%$						
Test for overall effect: Z = 0.63 (P = 0.53)						
Test for subgroup differences: $Chi^2 = 0.58$, df = 1 (P = 0.45), $I^2 = 0\%$						

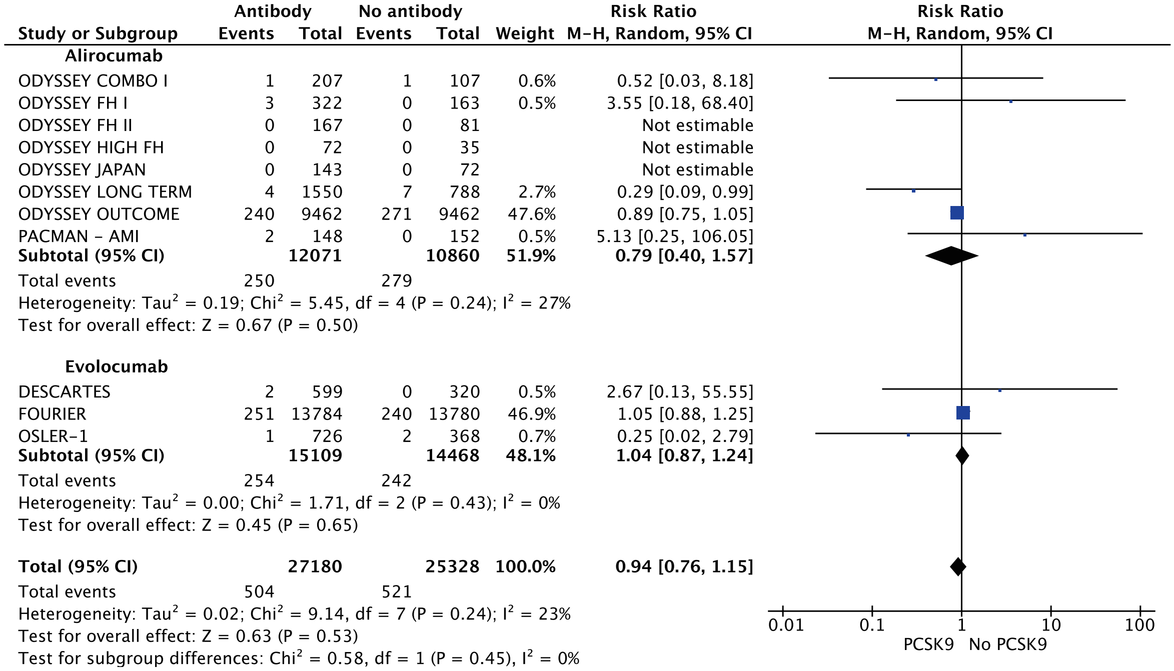

(B)

Study or Subgroup	Antibody Events	Antibody Total	No antibody Events	No antibody Total	Weight	Risk Ratio M-H, Fixed, 95% CI
**Alirocumab**						
ODYSSEY COMBO I	1	207	1	107	0.3%	0.52 [0.03, 8.18]
ODYSSEY FH I	3	322	0	163	0.1%	3.55 [0.18, 68.40]
ODYSSEY FH II	0	167	0	81		Not estimable
ODYSSEY HIGH FH	0	72	0	35		Not estimable
ODYSSEY JAPAN	0	143	0	72		Not estimable
ODYSSEY LONG TERM	4	1550	7	788	1.8%	0.29 [0.09, 0.99]
ODYSSEY OUTCOME	240	9462	271	9462	51.5%	0.89 [0.75, 1.05]
PACMAN – AMI	2	148	0	152	0.1%	5.13 [0.25, 106.05]
**Subtotal (95% CI)**		**12071**		**10860**	**53.7%**	**0.88 [0.74, 1.04]**
Total events	250		279			
Heterogeneity: $Chi^2 = 5.45$, df = 4 (P = 0.24); $I^2 = 27\%$						
Test for overall effect: Z = 1.52 (P = 0.13)						
**Evolocumab**						
DESCARTES	2	599	0	320	0.1%	2.67 [0.13, 55.55]
FOURIER	251	13784	240	13780	45.6%	1.05 [0.88, 1.25]
OSLER–1	1	726	2	368	0.5%	0.25 [0.02, 2.79]
**Subtotal (95% CI)**		**15109**		**14468**	**46.3%**	**1.04 [0.87, 1.24]**
Total events	254		242			
Heterogeneity: $Chi^2 = 1.71$, df = 2 (P = 0.43); $I^2 = 0\%$						
Test for overall effect: Z = 0.45 (P = 0.65)						
**Total (95% CI)**		**27180**		**25328**	**100.0%**	**0.95 [0.85, 1.08]**
Total events	504		521			
Heterogeneity: $Chi^2 = 9.14$, df = 7 (P = 0.24); $I^2 = 23\%$						
Test for overall effect: Z = 0.77 (P = 0.44)						
Test for subgroup differences: $Chi^2 = 1.91$, df = 1 (P = 0.17), $I^2 = 47.5\%$						

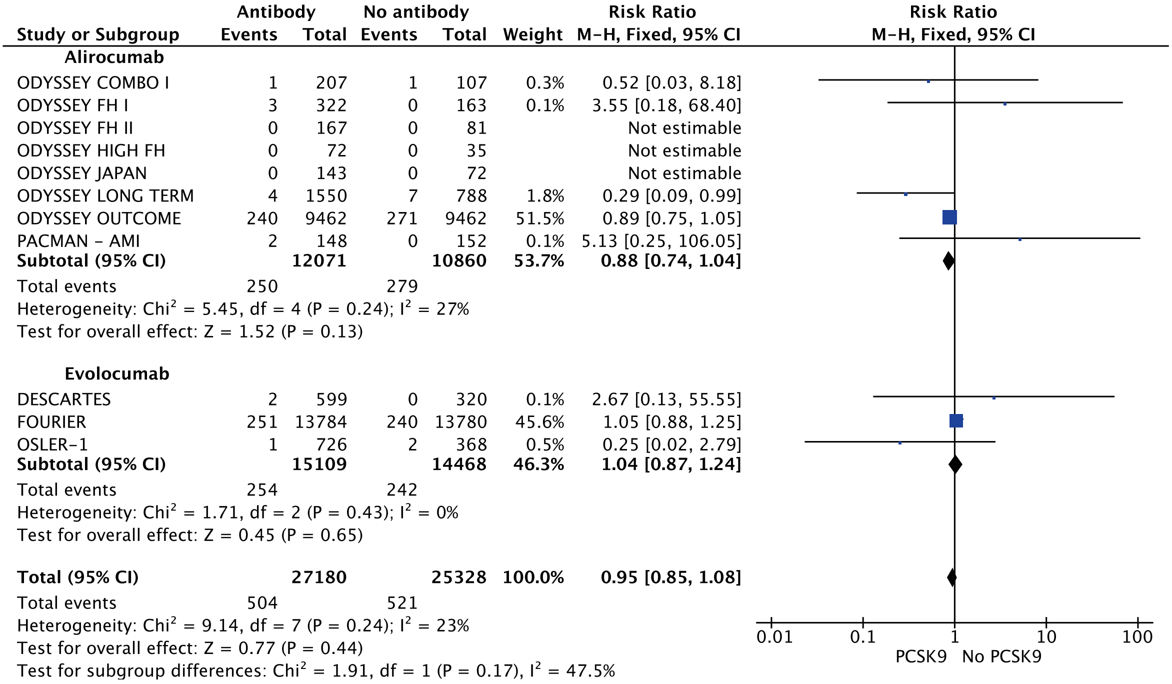

Supplementary Figure 5. Risk estimates using random effect (A) and fixed effect (B) models for cardiovascular mortality.

9 Supplementary Figure 6.

(A)

Study or Subgroup	Antibody Events	Antibody Total	No antibody Events	No antibody Total	Weight	Risk Ratio M-H, Random, 95% CI
**Alirocumab**						
ODYSSEY COMBO I	1	207	1	107	0.2%	0.52 [0.03, 8.18]
ODYSSEY FH I	1	322	1	163	0.2%	0.51 [0.03, 8.04]
ODYSSEY FH II	0	167	1	81	0.1%	0.16 [0.01, 3.95]
ODYSSEY HIGH FH	4	72	0	35	0.2%	4.44 [0.25, 80.21]
ODYSSEY JAPAN	1	143	1	72	0.2%	0.50 [0.03, 7.93]
ODYSSEY LONG TERM	14	1550	18	788	2.6%	0.40 [0.20, 0.79]
ODYSSEY OUTCOME	626	9462	722	9462	49.6%	0.87 [0.78, 0.96]
PACMAN - AMI	2	148	3	152	0.4%	0.68 [0.12, 4.04]
**Subtotal (95% CI)**		**12071**		**10860**	**53.4%**	**0.76 [0.57, 1.01]**
Total events	649		747			
Heterogeneity: $Tau^2 = 0.03$; $Chi^2 = 7.57$, df = 7 (P = 0.37); $I^2 = 7\%$						
Test for overall effect: Z = 1.88 (P = 0.06)						
**Evolocumab**						
DESCARTES	1	599	0	320	0.1%	1.60 [0.07, 39.29]
FOURIER	468	13784	639	13780	44.4%	0.73 [0.65, 0.82]
GLAGOV	10	484	14	484	2.0%	0.71 [0.32, 1.59]
OSLER-1	3	736	0	368	0.1%	3.50 [0.18, 67.67]
**Subtotal (95% CI)**		**15603**		**14952**	**46.6%**	**0.73 [0.65, 0.82]**
Total events	482		653			
Heterogeneity: $Tau^2 = 0.00$; $Chi^2 = 1.31$, df = 3 (P = 0.73); $I^2 = 0\%$						
Test for overall effect: Z = 5.24 (P < 0.00001)						
**Total (95% CI)**		**27674**		**25812**	**100.0%**	**0.78 [0.70, 0.88]**
Total events	1131		1400			
Heterogeneity: $Tau^2 = 0.00$; $Chi^2 = 12.37$, df = 11 (P = 0.34); $I^2 = 11\%$						
Test for overall effect: Z = 4.19 (P < 0.0001)						
Test for subgroup differences: $Chi^2 = 0.04$, df = 1 (P = 0.84), $I^2 = 0\%$						

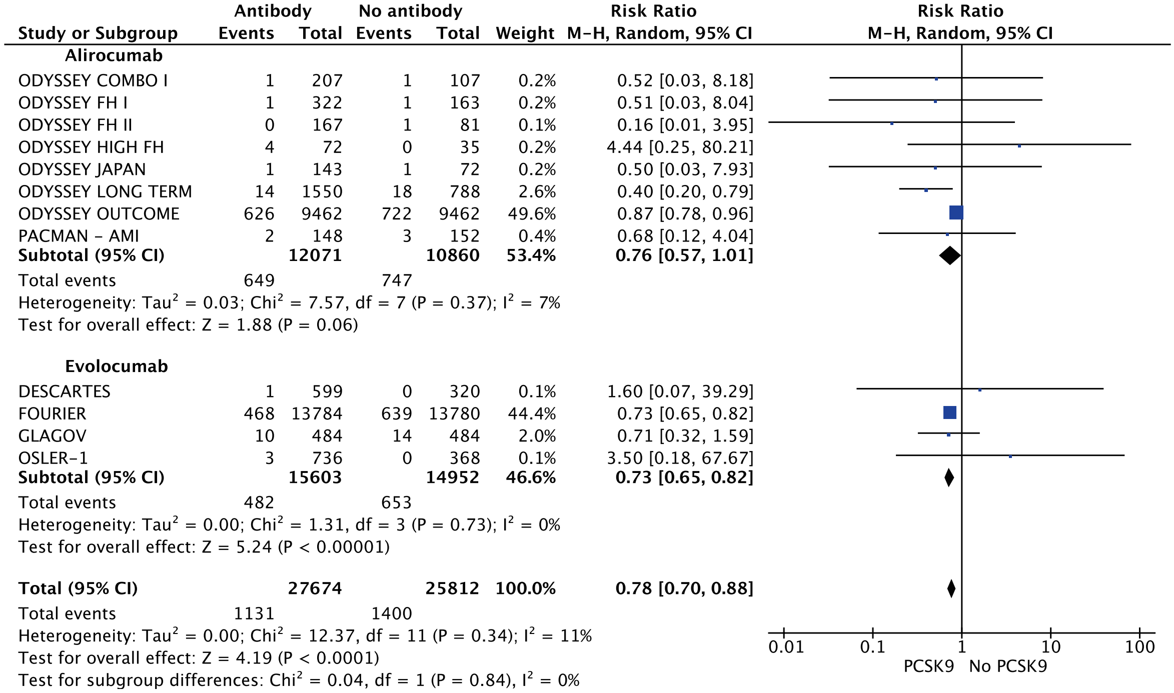

(B)

Study or Subgroup	Antibody Events	Antibody Total	No antibody Events	No antibody Total	Weight	Risk Ratio M-H, Fixed, 95% CI
**Alirocumab**						
ODYSSEY COMBO I	1	207	1	107	0.1%	0.52 [0.03, 8.18]
ODYSSEY FH I	1	322	1	163	0.1%	0.51 [0.03, 8.04]
ODYSSEY FH II	0	167	1	81	0.1%	0.16 [0.01, 3.95]
ODYSSEY HIGH FH	4	72	0	35	0.0%	4.44 [0.25, 80.21]
ODYSSEY JAPAN	1	143	1	72	0.1%	0.50 [0.03, 7.93]
ODYSSEY LONG TERM	14	1550	18	788	1.7%	0.40 [0.20, 0.79]
ODYSSEY OUTCOME	626	9462	722	9462	51.2%	0.87 [0.78, 0.96]
PACMAN - AMI	2	148	3	152	0.2%	0.68 [0.12, 4.04]
**Subtotal (95% CI)**		**12071**		**10860**	**53.6%**	**0.85 [0.77, 0.94]**
Total events	649		747			
Heterogeneity: $Chi^2 = 7.57$, df = 7 (P = 0.37); $I^2 = 7\%$						
Test for overall effect: Z = 3.13 (P = 0.002)						
**Evolocumab**						
DESCARTES	1	599	0	320	0.0%	1.60 [0.07, 39.29]
FOURIER	468	13784	639	13780	45.3%	0.73 [0.65, 0.82]
GLAGOV	10	484	14	484	1.0%	0.71 [0.32, 1.59]
OSLER-1	3	736	0	368	0.0%	3.50 [0.18, 67.67]
**Subtotal (95% CI)**		**15603**		**14952**	**46.4%**	**0.74 [0.66, 0.83]**
Total events	482		653			
Heterogeneity: $Chi^2 = 1.31$, df = 3 (P = 0.73); $I^2 = 0\%$						
Test for overall effect: Z = 5.22 (P < 0.00001)						
**Total (95% CI)**		**27674**		**25812**	**100.0%**	**0.80 [0.74, 0.86]**
Total events	1131		1400			
Heterogeneity: $Chi^2 = 12.37$, df = 11 (P = 0.34); $I^2 = 11\%$						
Test for overall effect: Z = 5.83 (P < 0.00001)						
Test for subgroup differences: $Chi^2 = 3.46$, df = 1 (P = 0.06), $I^2 = 71.1\%$						

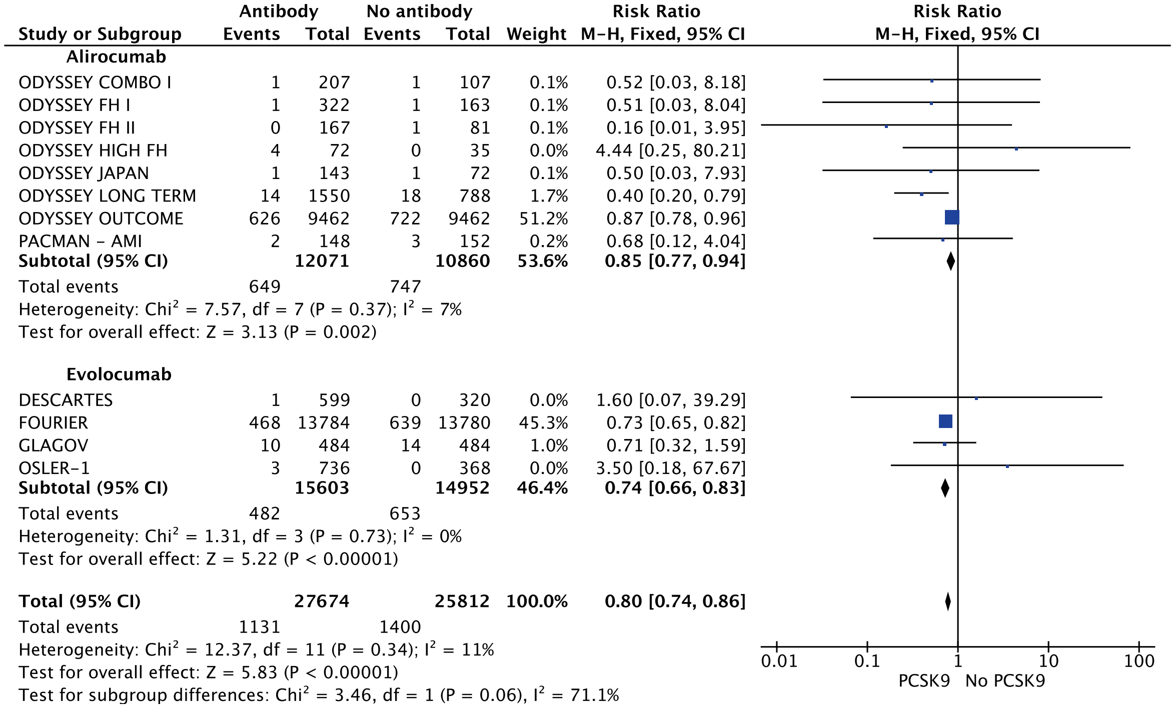

Supplementary Figure 6. Risk estimates using random effect (A) and fixed effect (B) models for myocardial infarction.

10 Supplementary Figure 7.

(A)

Study or Subgroup	Antibody Events	Antibody Total	No antibody Events	No antibody Total	Weight	Risk Ratio M-H, Random, 95% CI
**Alirocumab**						
ODYSSEY COMBO I	2	207	0	107	0.2%	2.60 [0.13, 53.60]
ODYSSEY FH I	1	322	0	163	0.2%	1.52 [0.06, 37.19]
ODYSSEY FH II	0	167	0	81		Not estimable
ODYSSEY HIGH FH	0	72	0	35		Not estimable
ODYSSEY JAPAN	0	143	0	72		Not estimable
ODYSSEY LONG TERM	9	1550	2	788	0.9%	2.29 [0.50, 10.56]
ODYSSEY OUTCOME	111	9462	152	9462	34.8%	0.73 [0.57, 0.93]
**Subtotal (95% CI)**		**11923**		**10708**	**36.1%**	**0.76 [0.60, 0.96]**
Total events	123		154			
Heterogeneity: $Tau^2 = 0.00$; $Chi^2 = 2.92$, df = 3 (P = 0.40); $I^2 = 0\%$						
Test for overall effect: Z = 2.26 (P = 0.02)						
**Evolocumab**						
FOURIER	207	13784	262	13780	62.9%	0.79 [0.66, 0.95]
GLAGOV	2	484	3	484	0.6%	0.67 [0.11, 3.97]
OSLER-1	3	736	1	368	0.4%	1.50 [0.16, 14.37]
**Subtotal (95% CI)**		**15004**		**14632**	**63.9%**	**0.79 [0.66, 0.95]**
Total events	212		266			
Heterogeneity: $Tau^2 = 0.00$; $Chi^2 = 0.34$, df = 2 (P = 0.84); $I^2 = 0\%$						
Test for overall effect: Z = 2.55 (P = 0.01)						
**Total (95% CI)**		**26927**		**25340**	**100.0%**	**0.78 [0.68, 0.90]**
Total events	335		420			
Heterogeneity: $Tau^2 = 0.00$; $Chi^2 = 3.33$, df = 6 (P = 0.77); $I^2 = 0\%$						
Test for overall effect: Z = 3.40 (P = 0.0007)						
Test for subgroup differences: $Chi^2 = 0.07$, df = 1 (P = 0.79), $I^2 = 0\%$						

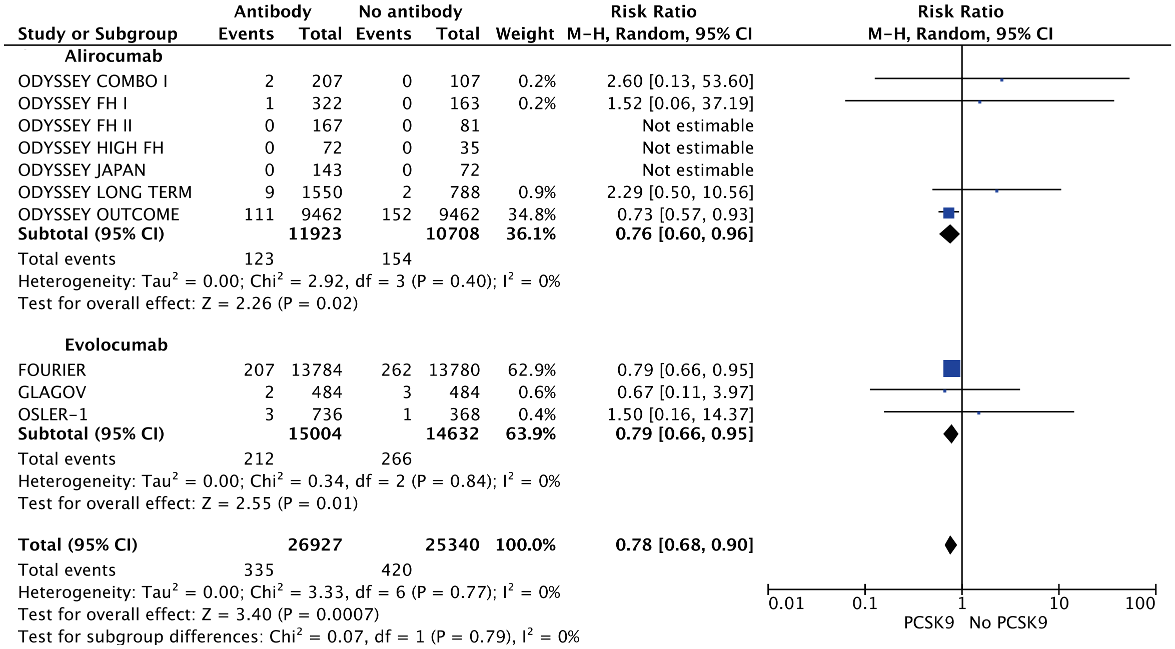

(8)

Study or Subgroup	Antibody Events	Antibody Total	No antibody Events	No antibody Total	Weight	Risk Ratio M-H, Fixed, 95% CI
**Alirocumab**						
ODYSSEY COMBO I	2	207	0	107	0.2%	2.60 [0.13, 53.60]
ODYSSEY FH I	1	322	0	163	0.2%	1.52 [0.06, 37.19]
ODYSSEY FH II	0	167	0	81		Not estimable
ODYSSEY HIGH FH	0	72	0	35		Not estimable
ODYSSEY JAPAN	0	143	0	72		Not estimable
ODYSSEY LONG TERM	9	1550	2	788	0.6%	2.29 [0.50, 10.56]
ODYSSEY OUTCOME	111	9462	152	9462	36.0%	0.73 [0.57, 0.93]
**Subtotal (95% CI)**		**11923**		**10708**	**36.9%**	**0.77 [0.61, 0.97]**
Total events	123		154			
Heterogeneity: $Chi^2 = 2.92$, df = 3 (P = 0.40); $I^2 = 0\%$						
Test for overall effect: Z = 2.18 (P = 0.03)						
**Evolocumab**						
FOURIER	207	13784	262	13780	62.0%	0.79 [0.66, 0.95]
GLAGOV	2	484	3	484	0.7%	0.67 [0.11, 3.97]
OSLER-1	3	736	1	368	0.3%	1.50 [0.16, 14.37]
**Subtotal (95% CI)**		**15004**		**14632**	**63.1%**	**0.79 [0.66, 0.95]**
Total events	212		266			
Heterogeneity: $Chi^2 = 0.34$, df = 2 (P = 0.84); $I^2 = 0\%$						
Test for overall effect: Z = 2.55 (P = 0.01)						
**Total (95% CI)**		**26927**		**25340**	**100.0%**	**0.78 [0.68, 0.90]**
Total events	335		420			
Heterogeneity: $Chi^2 = 3.33$, df = 6 (P = 0.77); $I^2 = 0\%$						
Test for overall effect: Z = 3.35 (P = 0.0008)						
Test for subgroup differences: $Chi^2 = 0.04$, df = 1 (P = 0.84), $I^2 = 0\%$						

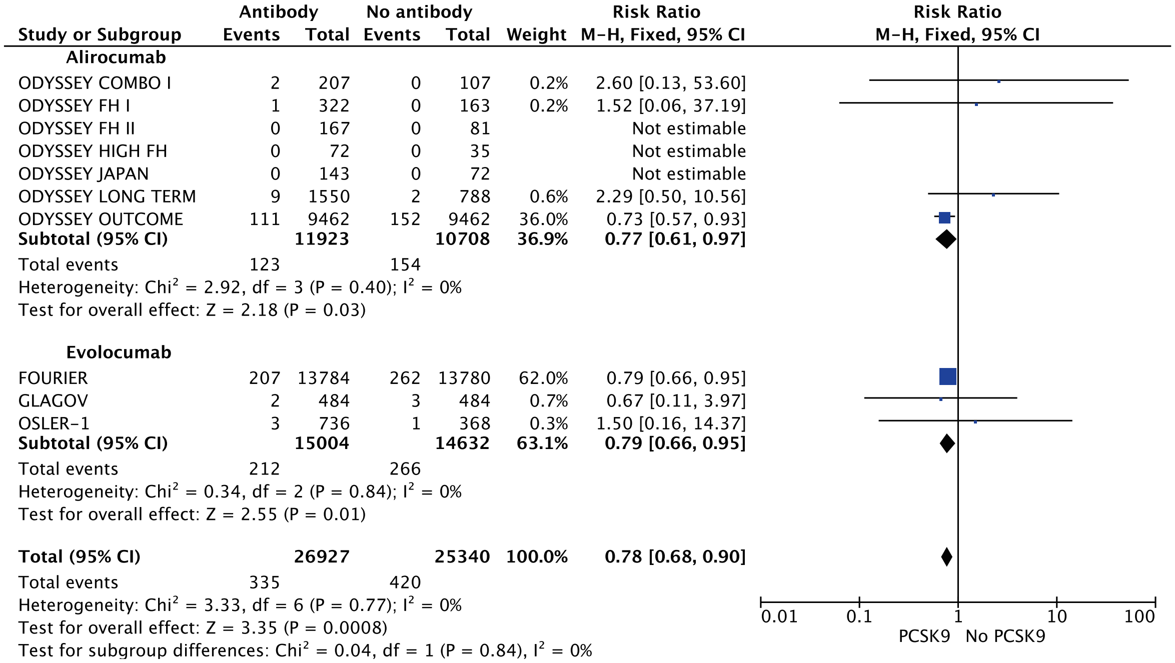

Supplementary Figure 7. Risk estimates using random effect (A) and fixed effect (B) models for stroke.

11 Supplementary Figure 8.

(A)

Study or Subgroup	Antibody Events	Antibody Total	No antibody Events	No antibody Total	Weight	Risk Ratio M-H, Random, 95% CI
**Alirocumab**						
ODYSSEY COMBO I	3	207	1	107	0.1%	1.55 [0.16, 14.73]
ODYSSEY FH I	2	322	2	163	0.1%	0.51 [0.07, 3.56]
ODYSSEY FH II	2	167	1	81	0.1%	0.97 [0.09, 10.54]
ODYSSEY HIGH FH	5	72	0	35	0.0%	5.42 [0.31, 95.43]
ODYSSEY JAPAN	2	143	1	72	0.1%	1.01 [0.09, 10.92]
ODYSSEY LONG TERM	48	1550	24	788	1.8%	1.02 [0.63, 1.65]
ODYSSEY OUTCOME	731	9462	828	9462	45.0%	0.88 [0.80, 0.97]
PACMAN - AMI	12	148	28	152	1.0%	0.44 [0.23, 0.83]
**Subtotal (95% CI)**		**12071**		**10860**	**48.1%**	**0.88 [0.80, 0.96]**
Total events	805		885			
Heterogeneity: $Tau^2 = 0.00$; $Chi^2 = 7.00$, df = 7 (P = 0.43); $I^2 = 0\%$						
Test for overall effect: Z = 2.81 (P = 0.005)						
**Evolocumab**						
FOURIER	759	13784	965	13780	48.2%	0.79 [0.72, 0.86]
GLAGOV	50	484	66	484	3.4%	0.76 [0.54, 1.07]
OSLER-1	6	736	4	368	0.3%	0.75 [0.21, 2.64]
**Subtotal (95% CI)**		**15004**		**14632**	**51.9%**	**0.78 [0.72, 0.86]**
Total events	815		1035			
Heterogeneity: $Tau^2 = 0.00$; $Chi^2 = 0.05$, df = 2 (P = 0.98); $I^2 = 0\%$						
Test for overall effect: Z = 5.37 (P < 0.00001)						
**Total (95% CI)**		**27075**		**25492**	**100.0%**	**0.83 [0.78, 0.88]**
Total events	1620		1920			
Heterogeneity: $Tau^2 = 0.00$; $Chi^2 = 9.94$, df = 10 (P = 0.45); $I^2 = 0\%$						
Test for overall effect: Z = 5.81 (P < 0.00001)						
Test for subgroup differences: $Chi^2 = 2.89$, df = 1 (P = 0.09), $I^2 = 65.4\%$						

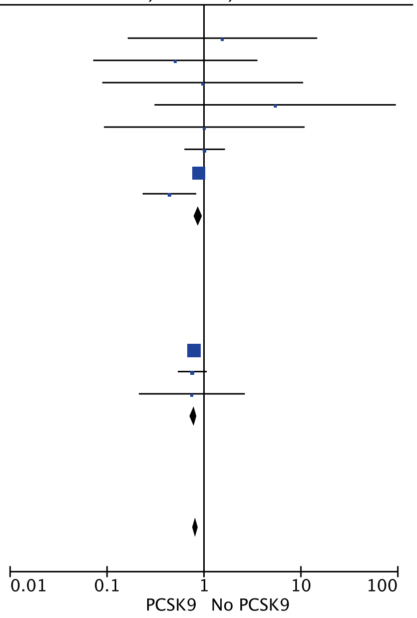

(B)

Study or Subgroup	Antibody Events	Antibody Total	No antibody Events	No antibody Total	Weight	Risk Ratio M-H, Fixed, 95% CI
**Alirocumab**						
ODYSSEY COMBO I	3	207	1	107	0.1%	1.55 [0.16, 14.73]
ODYSSEY FH I	2	322	2	163	0.1%	0.51 [0.07, 3.56]
ODYSSEY FH II	2	167	1	81	0.1%	0.97 [0.09, 10.54]
ODYSSEY HIGH FH	5	72	0	35	0.0%	5.42 [0.31, 95.43]
ODYSSEY JAPAN	2	143	1	72	0.1%	1.01 [0.09, 10.92]
ODYSSEY LONG TERM	48	1550	24	788	1.6%	1.02 [0.63, 1.65]
ODYSSEY OUTCOME	731	9462	828	9462	42.9%	0.88 [0.80, 0.97]
PACMAN - AMI	12	148	28	152	1.4%	0.44 [0.23, 0.83]
**Subtotal (95% CI)**		**12071**		**10860**	**46.3%**	**0.88 [0.80, 0.96]**
Total events	805		885			
Heterogeneity: $Chi^2 = 7.00$, df = 7 (P = 0.43); $I^2 = 0\%$						
Test for overall effect: Z = 2.78 (P = 0.005)						
**Evolocumab**						
FOURIER	759	13784	965	13780	50.0%	0.79 [0.72, 0.86]
GLAGOV	50	484	66	484	3.4%	0.76 [0.54, 1.07]
OSLER-1	6	736	4	368	0.3%	0.75 [0.21, 2.64]
**Subtotal (95% CI)**		**15004**		**14632**	**53.7%**	**0.78 [0.72, 0.86]**
Total events	815		1035			
Heterogeneity: $Chi^2 = 0.05$, df = 2 (P = 0.98); $I^2 = 0\%$						
Test for overall effect: Z = 5.36 (P < 0.00001)						
**Total (95% CI)**		**27075**		**25492**	**100.0%**	**0.83 [0.78, 0.88]**
Total events	1620		1920			
Heterogeneity: $Chi^2 = 9.94$, df = 10 (P = 0.45); $I^2 = 0\%$						
Test for overall effect: Z = 5.81 (P < 0.00001)						
Test for subgroup differences: $Chi^2 = 2.97$, df = 1 (P = 0.08), $I^2 = 66.3\%$						

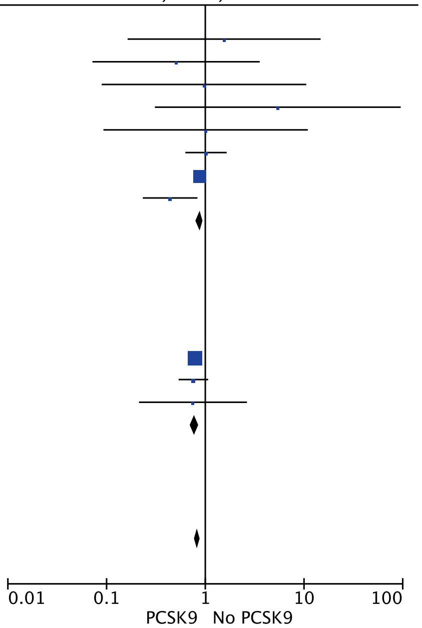

Supplementary Figure 8. Risk estimates using random effect (A) and fixed effect (B) models for coronary revascularization.

12 Supplementary Figure 9.

(A)

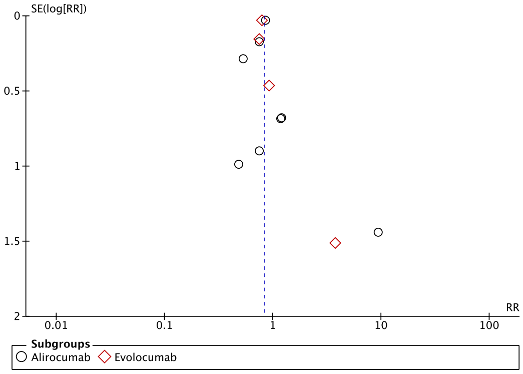

(B)

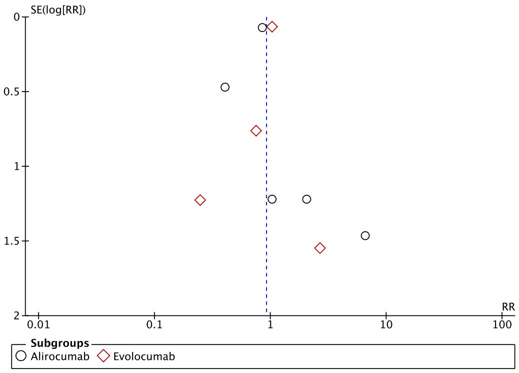

(C)

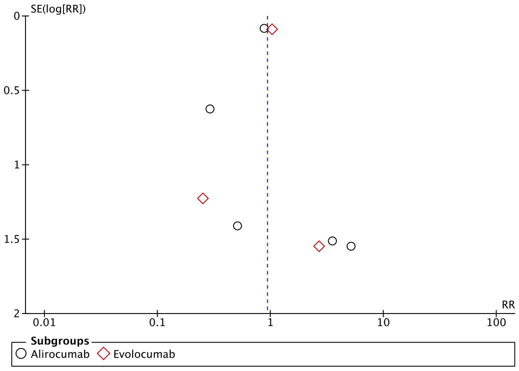

(D)

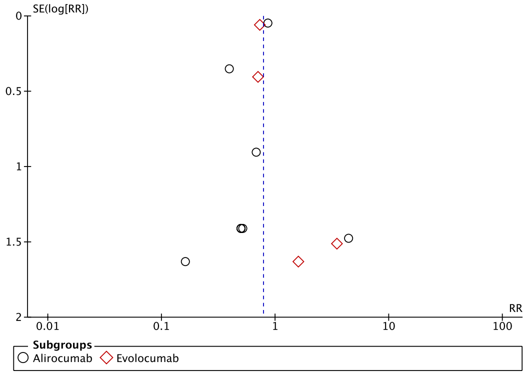

(E)

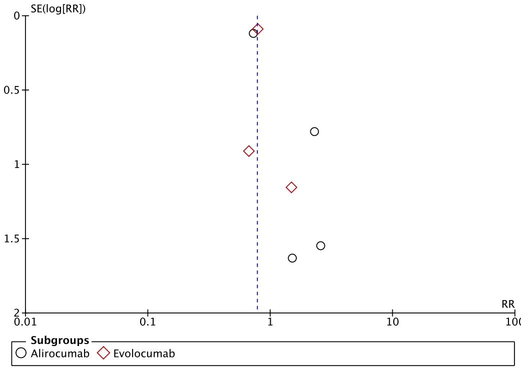

(F)

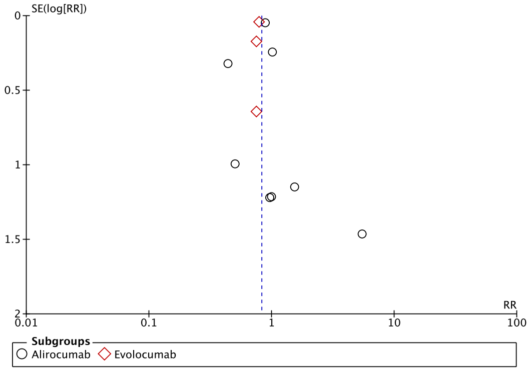

Supplementary Figure 9: Funnel plot analysis for major vascular events (A), all-cause death (B), cardiovascular mortality (C), myocardial infarction (D), stroke (E), and coronary revascularization (F).

13 Supplementary Figure 10.

(A)

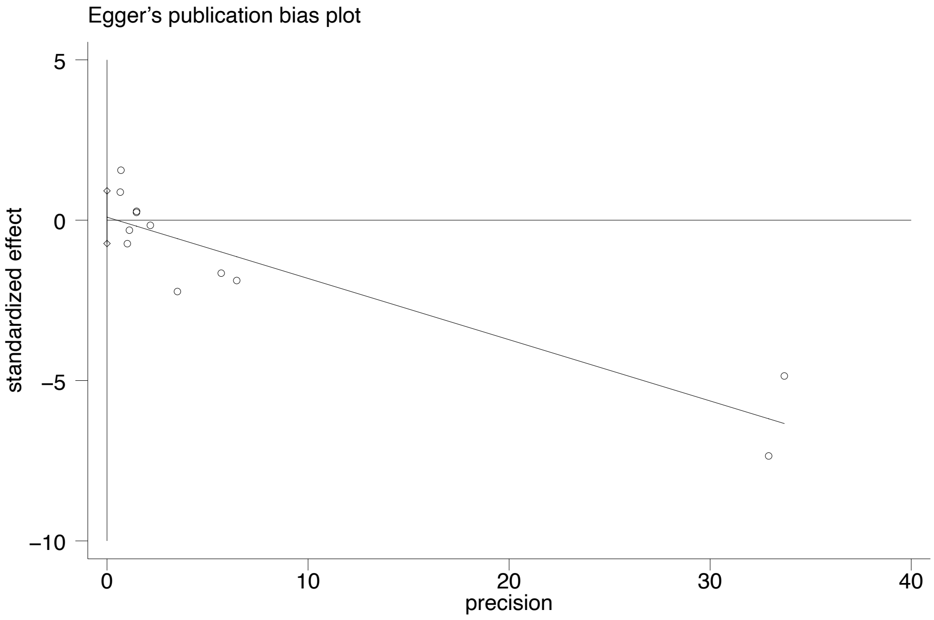

(B)

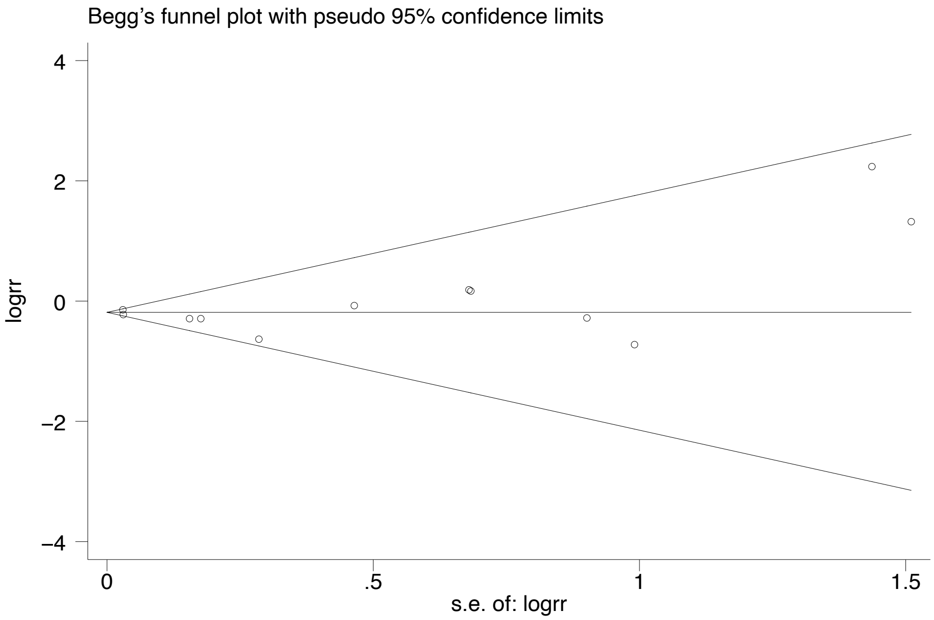

Supplementary Figure 10: Egger's test (A), Begg's test (B).
